# MagFRET: The First Genetically Encoded Fluorescent Mg^2+^ Sensor

**DOI:** 10.1371/journal.pone.0082009

**Published:** 2013-12-02

**Authors:** Laurens H. Lindenburg, Jan L. Vinkenborg, Jorn Oortwijn, Stijn J. A. Aper, Maarten Merkx

**Affiliations:** Laboratory of Chemical Biology, Department of Biomedical Engineering, Eindhoven University of Technology, Eindhoven, The Netherlands; Instituto de Tecnologica Química e Biológica, UNL, Portugal

## Abstract

Magnesium has important structural, catalytic and signaling roles in cells, yet few tools exist to image this metal ion in real time and at subcellular resolution. Here we report the first genetically encoded sensor for Mg^2+^, MagFRET-1. This sensor is based on the high-affinity Mg^2+^ binding domain of human centrin 3 (HsCen3), which undergoes a transition from a molten-globular apo form to a compactly-folded Mg^2+^-bound state. Fusion of Cerulean and Citrine fluorescent domains to the ends of HsCen3, yielded MagFRET-1, which combines a physiologically relevant Mg^2+^ affinity (*K*_d_ = 148 µM) with a 50% increase in emission ratio upon Mg^2+^ binding due to a change in FRET efficiency between Cerulean and Citrine. Mutations in the metal binding sites yielded MagFRET variants whose Mg^2+^ affinities were attenuated 2- to 100-fold relative to MagFRET-1, thus covering a broad range of Mg^2+^ concentrations. *In situ* experiments in HEK293 cells showed that MagFRET-1 can be targeted to the cytosol and the nucleus. Clear responses to changes in extracellular Mg^2+^ concentration were observed for MagFRET-1-expressing HEK293 cells when they were permeabilized with digitonin, whereas similar changes were not observed for intact cells. Although MagFRET-1 is also sensitive to Ca^2+^, this affinity is sufficiently attenuated (*K*_d_ of 10 µM) to make the sensor insensitive to known Ca^2+^ stimuli in HEK293 cells. While the potential and limitations of the MagFRET sensors for intracellular Mg^2+^ imaging need to be further established, we expect that these genetically encoded and ratiometric fluorescent Mg^2+^ sensors could prove very useful in understanding intracellular Mg^2+^ homeostasis and signaling.

## Introduction

Magnesium is the most abundant intracellular divalent cation and is involved in numerous essential cellular processes including replication, transcription, translation and energy metabolism. In addition to its omnipresent role as an essential enzymatic cofactor, Mg^2+^ is also important for chromatin stability and regulates specific ion channels [1], [2]. The total cellular Mg^2+^ concentration ranges between 14–20 mM, but the concentration of free Mg^2+^ in the cytosol has been estimated to be between 0.1 and 1.5 mM [3]–[7]. Given its importance to so many different cellular processes, the intracellular Mg^2+^ concentration is generally believed to be strongly buffered and tightly regulated by the combined action of magnesium binding (macro)molecules (proteins, ribonucleic acids, ATP, etc.), storage in organelles and the action of Mg^2+^ channels [8]–[10]. Hereditary disorders related to Mg^2+^ homeostasis have been shown to result in diminished kidney functioning and in severe cases to renal failure, muscle spasms and seizures [11]. Magnesium deficiency has also been shown to accelerate cellular senescence [12], providing a potential link between low dietary magnesium intake and the early onset of aging diseases such as diabetes [13], cardiovascular diseases [14] and osteoporosis [15]. Recent studies suggested that T cell activation following antigen receptor stimulation was dependent on a transient influx of Mg^2+^ in the cytosol, implicating a novel role for Mg^2+^ as second messenger in intracellular signal transduction [16].

Despite the abundance and importance of Mg^2+^, the intracellular regulation of Mg^2+^ homeostasis and the putative role of Mg^2+^ in intracellular signal transduction are not well understood. In part this is because of a lack of convenient molecular tools to image the intracellular Mg^2+^ concentration in single living cells in real time [17]. Magnesium-selective microelectrodes have been used to determine cytosolic Mg^2+^ levels in different muscle cells, revealing concentrations between 0.7 and 0.9 mM [4]. However, these microelectrodes are highly invasive and do not provide spatial information. Another method to probe the intracellular concentration of Mg^2+^ is the measurement of the ratio of Mg^2+^-bound and Mg^2+^-free ATP using ^31^P NMR [18]. While non-invasive, ^31^P NMR measures Mg^2+^ indirectly and averaged over a large collection of cells [19], [20]. The currently most commonly applied approach uses synthetic dyes that alter their fluorescent properties upon binding of Mg^2+^ [21]–[27]. However, many of the available dyes show limited specificity for Mg^2+^ and often bind Ca^2+^ with low micromolar affinity [22], [25], [26], which has been shown to interfere in an intracellular setting [28]. A notable exception is KMG-104 and related dyes developed by Kuzuki and coworkers, whose affinity for Mg^2+^ is higher than for Ca^2+^ (*K*_d_ = 2.1 and 7.5 mM, respectively), rendering these dyes completely insensitive to physiological changes in cytosolic Ca^2+^ concentration [24], [29], [30]. Recently a variant of this dye, KMG-103 was reported that showed preferred accumulation in mitochondria [27]. Like most synthetic Mg^2+^ dyes, the KMG dyes are intensiometric, making Mg^2+^ quantification challenging and sensitive to changes in sensor concentration. A few ratiometric Mg^2+^ fluorescent dyes (e.g. Mag-Fura and Mag-Indo) exist, yet these have the disadvantage that they require potentially cytotoxic UV excitation [17].

Genetically encoded fluorescent sensor proteins provide an attractive alternative to small-molecule fluorescent sensors, because they do not require cell-invasive procedures, their concentration can be tightly controlled and they can be targeted to different locations in the cell [31]. Many of these sensors consist of metal binding domain(s) fused to a donor and an acceptor fluorescent domain capable of Förster Resonance Energy Transfer (FRET). Modulation of the distance and/or orientation of the fluorescent domains following metal binding affects the FRET efficiency, which can be detected as change in the emission ratio, an output signal that is independent of sensor concentration. In addition, the use of natural metal binding protein domains often ensures a physiologically relevant metal binding affinity and specificity. The wealth of genetically encoded sensors that have been developed for Ca^2+^ [32]–[35], and more recently also for Zn^2+^ [36]–[39] and Cu^+^ [40], [41], have made important contributions to the understanding of intracellular metal homeostasis and signaling.

Surprisingly, no genetically encoded sensors have thus far been reported for Mg^2+^. One of the specific challenges in this case is metal binding specificity. Mg^2+^ and Ca^2+^ show similar coordination chemistry and often bind to the same metal binding proteins, with Ca^2+^ typically showing stronger binding. Here we report the first genetically encoded fluorescent sensor (MagFRET-1) for Mg^2+^ by taking advantage of the particular metal binding properties of the N-terminal part of the HsCen3 protein, which binds both Ca^2+^ and Mg^2+^ with high affinity [42]. We show that Mg^2+^ binding to MagFRET-1 induces folding from a molten-globule state that results in an increase in FRET. Mutagenesis of metal binding site residues allowed further tuning of the metal binding properties, yielding MagFRET variants with *K*_d_ values for Mg^2+^ binding ranging between 0.15 and 15 mM. While also responsive to Ca^2+^ *in vitro*, we show that the Ca^2+^ affinities of the MagFRET sensors are sufficiently attenuated that they are not responsive to normal Ca^2+^ fluctuations *in situ*.

## Materials and Methods

### Cloning of expression plasmids

DNA encoding the N-terminal fragment of HsCen3 (residues 23 to 98 [42]) was obtained as a synthetic pUC57 construct (GenScript, USA). Restriction of this construct with restriction enzymes *NheI* and *NcoI* yielded an insert fragment that was compatible with a pET28a acceptor vector encoding for His_6_-Cerulean-(GGS)_18_-Citrine [43] that had been treated with restriction enzymes *SpeI* (creating an *NheI*-compatible cohesive overhang) and *NcoI*. A ligation was carried out at equimolar vector-to-insert ratio using T4 DNA ligase (TaKaRA Mighty Mix, Takara, USA) at 16°C for 1 hour following the manufacturer's instructions, resulting in pET28a-MagFRET-1 (**Figure S1**). The *SpeI* restriction site in the pET28a-His_6_-Cerulean-(GGS)_18_-Citrine acceptor vector was located 8 residues upstream of the Cerulean C-terminus, such that in the final MagFRET construct, the native flexible C-terminus of Cerulean was deleted, resulting in tighter allosteric coupling between changes in HsCen3 conformation and changes in the fluorescent domains' interchromophore distance. The mammalian expression vector for MagFRET-1 was obtained by digesting pET28a-MagFRET-1 using restriction enzymes *AgeI* and *NotI*. Ligation into a peCALWY-1 vector [36] that was digested with the same restriction enzymes resulted in pCMV-MagFRET-1 (**Figure** **S2**). Mutations in metal binding loop I and II of MagFRET-1 were introduced using site-directed mutagenesis (QuikChange Multi Site-Directed Mutagenesis Kit for mutations in loop I and QuikChange Site-Directed Mutagenesis Kit for mutations in loop II), following the kit manufacturer's (Qiagen) instructions. Primers used to introduce these mutations are listed in **Table S1**. To obtain the mammalian expression vector encoding for a nuclear-targeted MagFRET-1 (MagFRET-1-NLS), a pUC57 vector containing a synthetic gene encoding for the final part of Citrine together with three PKKKRKV repeats was digested using restriction enzymes *HindIII* and *NotI*, followed by ligation into a pCMV-MagFRET-1 plasmid that was treated with the same restriction enzymes (**Figure S3**). The correct open reading frame of each sensor was confirmed by Sanger dideoxy sequencing (Baseclear, Leiden, The Netherlands).

### Protein expression and purification

*E. coli* BL21(DE3) cells were used for protein expression. A single colony was used to inoculate 5 mL LB medium (10 g/L NaCl, 10 g/L peptone, 5 g/L yeast extract) supplemented with 30 µg/mL kanamycin which was grown overnight at 225 rpm at 37°C. Overnight cultures were diluted in 500 mL LB medium containing kanamycin (30 µg/mL) and grown until an optical density of 0.6–0.8 was reached at 600 nm wavelength. Protein expression was induced by the addition of 0.1 mM isopropyl β-D-1-thiogalactopyranoside (IPTG, Sigma). Bacteria were cultured overnight at 225 rpm at 25°C and harvested by centrifugation at 10,000 g for 10 minutes at 4°C. The cell pellets were lysed using Bugbuster reagent (Novagen) according to the manufacturer's instructions. The resulting soluble protein fraction was used for further purification. The expressed MagFRET proteins contain an N-terminal hexahistidine-tag. Ni^2+^-NTA resin (His-bind, Novagen) was used for affinity chromatography following the manufacturer's instructions. After elution of the protein using 0.5 M imidazole, the protein was dialyzed overnight against 100 volumes of 20 mM Tris-HCl (pH 8.4), 150 mM NaCl and 2.5 mM CaCl_2_ using a 12–14 kDa Molecular Weight Cut-Off (MWCO) dialysis membrane (Spectropore) at 4°C. The hexahistidine-tag was subsequently removed by the addition of 0.3 U thrombin protease (Novagen) per mg protein at a 0.2 mg/mL protein concentration and incubated for 24 hours at 4°C. His-tags and uncleaved proteins were removed using Ni^2+^ affinity chromatography. The flow-through was further purified using size exclusion chromatography (SEC) on a Sephacryl S-200 High resolution column (GE Healthcare). Fractions containing pure proteins were pooled, concentrated, frozen in liquid nitrogen and stored in aliquots at −80°C. The purity and correct molecular weight of the obtained proteins was confirmed by sodium dodecyl sulfate polyacrylamide gel electrophoresis (SDS-PAGE, with 12% acrylamide) analysis. The protein concentration was determined using the absorption at 515 nm (ND-1000 Nanodrop) and a molar extinction coefficient of 77,000 M^−1^cm^−1^ for Citrine [44].

### Fluorescence spectroscopy

Unless otherwise mentioned, magnesium and calcium titrations were performed in 150 mM Hepes (4-(2-hydroxyethyl)-1-piperazineethanesulfonic acid) (pH 7.1), 100 mM NaCl, 10% (v/v) glycerol. Fluorescence emission spectra were recorded between 450 and 600 nm at a 0.2 µM protein concentration on a Varian Cary Eclipse fluorometer with an excitation wavelength of 420 nm. MgCl_2_ and CaCl_2_ (both from Sigma) were added at increasing concentrations from a concentrated stock solution in water. To determine the MagFRET-1 dissociation constant (*K_d_*) for Mg^2+^, the emission ratio (*R*) as a function of MgCl_2_ concentration ([*Mg^2+^*]) was fit to equation 1, 
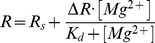
(1)where *R_S_* is the starting emission ratio in absence of Mg^2+^ and Δ*R* the difference in emission ratio between the Mg^2+^-free and Mg^2+^-saturated form of MagFRET-1. To determine the MagFRET *K_d_*_1_ and *K_d_*_2_ values associated with the first and second Ca^2+^-binding events respectively, the emission ratio (*R*) as a function of CaCl_2_ concentration ([*Ca* ^2+^]) was fit to a double binding event using equation 2, 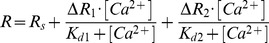
(2)where *R_S_* is the starting emission ratio in absence of Ca^2+^, Δ*R*_1_ is the difference between the emission ratio in the Ca^2+^-free state and the state in which a single Ca^2+^ ion has bound to the first metal binding loop of HsCen3, Δ*R*_2_ the difference between the latter state and the state in which a second Ca^2+^ ion has bound to EF-hand II. For the metal specificity measurements, either BaCl_2_, NiSO_4_, CuSO_4_, ZnCl_2_ or FeCl_3_ was added from 1000× concentration stock solutions to a final concentration of 10 µM, followed by addition of 1 mM MgCl_2_. For titrations (with MgCl_2_, NaCl and ammonium acetate) testing the effect of ionic strength on the sensor, a low salt buffer was used, consisting of 20 mM Hepes (pH 7.1), 10 mM NaCl and 10% (v/v) glycerol. To test for pH sensitivity of MagFRET-1, MgCl_2_ and CaCl_2_ titrations were carried out in buffers where Hepes was replaced with either MES (2-(N-morpholino)ethanesulfonic acid) or Tris (tris(hydroxymethyl)aminomethane) in the standard measurement buffer. The MES-containing buffer was prepared at pH 6 while the Tris-containing buffer was prepared at pH 8.

### Cell culturing and transfection

HEK293 cells were grown in Dulbecco's modified Eagle medium (DMEM, Sigma) containing 10% (vol/vol) fetal bovine serum (FBS, Life Technologies), 3 mM glucose, 2 mM glutamine, 100 units mL^−1^ penicillin and 100 µg mL^−1^ streptomycin at 37°C and 5% CO_2_. Cells were plated on poly-L-lysine (Sigma) treated glass coverslips and transfected with 1.5 µg of plasmid DNA and 5 µg polyethyleneimine (PEI). Cells were imaged for transient expression 2 days after transfection. In addition to the pCMV-MagFRET-1 and pCMV-MagFRET-1-NLS constructs described above, cells were also transfected with peZinCh-NB [36], which encodes for the Cerulean-linker-Citrine protein that was used as a negative control. Details of the Western blot procedure are provided in Method S1.

### Fluorescence microscopy

To demonstrate correct localization and subcellular targeting of MagFRET-1, confocal microscopy (Leica TCS SP5 X) was used to image the sensor with high spatial resolution. Samples were excited using a 405 nm laser and emission was detected using a hybrid APD/PMT detector (HyD, Leica). Spectral emission windows were set to 460–490 nm for the Cerulean channel and 510–550 nm for the Citrine channel, using an acousto optical beam splitter (AOBS, Leica). Widefield fluorescence microscopy for FRET measurements was performed on an Axio observer D.1 (Zeiss) equipped with an Axiocam MRm monochrome digital camera (Zeiss) using Axiovision 4.7 software. Samples were excited using a HXP 120 Mercury lamp (Zeiss) and Cerulean and Citrine emission was recorded sequentially using filter set 47 (excitation BP 436/20, dichroic 455, emission BP 480/40) and 48 (excitation BP 436/20, dichroic 455, emission BP 535/30) (Zeiss) in a motorized filter turret. Emission of Oregon Green-BAPTA was recorded using filterset 38 HE (excitation 470/40, dichroic 495, emission BP 525/50) (Zeiss). Images were acquired using an apochromat 40× objective, with an exposure time of 200–300 ms.

### Imaging of response to changes in intracellular Mg^2+^

Prior to addition of Mg^2+^ or EDTA, cells were permeabilized by a 6 minute incubation of HEK293 cells in 400 µL intracellular buffer (IB) containing 10 µg/ml digitonin (Sigma). IB comprised 20 mM Hepes (pH 7.05), 140 mM KCl, 10 mM KH_2_PO_4_, 100 µM ATP, 2 mM Na^+^ succinate and 5.5 mM glucose. After 6 minutes, recordings were started and buffers containing increasing concentrations of EDTA or MgCl_2_ were added as stated in the main text. When adding MgCl_2_, KCl concentrations were reduced accordingly to maintain the Cl^−^ concentration at 140 mM. Imaging frequency was 0.1 Hz.

### Intracellular Ca2+ specificity

Intracellular calcium specificity measurements were performed on HEK293 cells transfected as described above. Modified Krebs-bicarbonate buffer was used, consisting of 10 mM Hepes (pH 7.4), 140 mM NaCl, 3.6 mM KCl, 0.5 mM NaH_2_PO_4_, 1.5 mM CaCl_2_, 25 mM NaHCO_3_ and 3 mM glucose. Where indicated, PAR-1 agonist peptide (sequence SFLLRN, Genscript, USA) or ATP (Sigma) were added to a final concentration of 50 µM. Control experiments were performed using non-transfected HEK293 cells that were loaded with 10 µM Oregon Green-BAPTA-AM (Life Technologies, Netherlands) in phosphate buffered saline (PBS) with 0.01% (w/v) Pluronic F-127 (Life Technologies) for 30 minutes. At the end of each experiment in which Oregon Green-BAPTA-AM was used, 20 µM of calcium ionophore A23187 (Sigma) was added. Imaging frequency was 0.2 Hz.

## Results

### Sensor design

**Figure 1:**
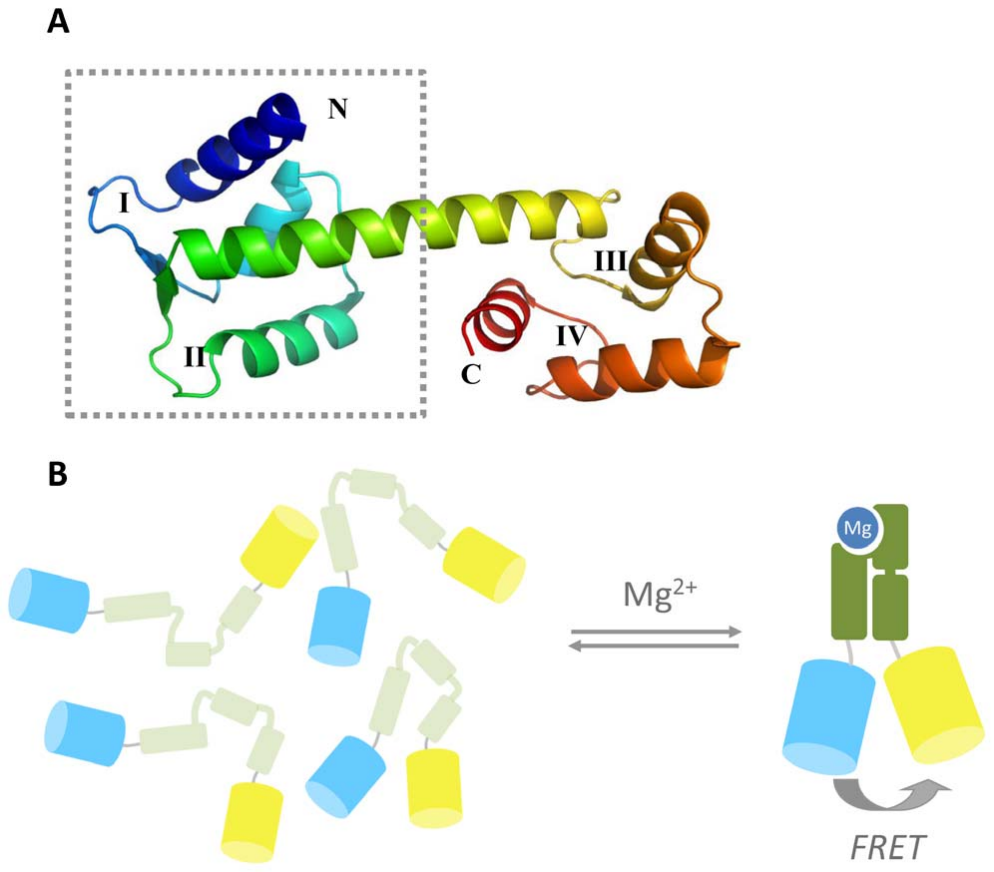
Design of the genetically encoded magnesium FRET sensor MagFRET. (A) Crystal structure (PDB code 2GGM) of HsCen2 in the calcium-bound, compact state. The typical helix-loop-helix structure can be observed, with EF-hands indicated by Roman numerals. The dotted lines indicate the N-terminal truncated part of the domain used in the sensor. In HsCen3, the high-affinity Mg^2+^/Ca^2+^ binding site is in loop I, and a much weaker Ca^2+^-binding site is found in loop II. (B) Schematic representation of MagFRET, where the N-terminal truncation of HsCen3 is flanked by Cerulean and Citrine. In absence of Mg^2+^, the HsCen3 domain is in a molten globule-like state, with little tertiary structure and a relatively large average distance between the fluorescent domains. Mg^2+^-binding induces a compact, well-defined tertiary structure, resulting in increased energy transfer between Cerulean and Citrine.

The construction of a FRET sensor for Mg^2+^ requires the availability of a metal binding domain that undergoes a large conformational change and displays a relatively high affinity for Mg^2+^ compared to Ca^2+^. Cox and coworkers previously reported that a truncated version of HsCen3 containing the first two of its four native EF-hand metal binding sites, undergoes a dramatic change in conformation upon metal binding from a molten globular (MG) state to a compact, natively-folded state [42]. Unlike most other EF hand-like proteins, which typically bind Ca^2+^ orders of magnitude more strongly than Mg^2+^, HsCen3's first EF hand is a high-affinity mixed Mg^2+^/Ca^2+^ binding site, with a reported *K*_d_ for Mg^2+^ of 10–28 µM and a *K*_d_ for Ca^2+^ of 1.5–8 µM. The second metal binding site was reported to bind only Ca^2+^, but with a much weaker affinity (*K*_d_ = 140 µM). HsCen3 is one of the four isoforms of human Centrin, a family of proteins that is involved in centriole duplication. We based our design on the structure of HsCen2, which shows high homology to HsCen3 and is the only isoform for which an X-ray structure has been determined (**Figure 1A**). The 11 kDa N-terminal fragment studied by Cox and coworkers contained the complete α-helix connecting the 2^nd^ and 3^rd^ EF hand sites. To decrease the distance between the N- and C-termini of the receptor part in the Mg^2+^-bound state, we decided to truncate this helix to approximately half its size (aa 23–98) and fuse it to the fluorescent proteins Cerulean and Citrine (**Figure 1B**). To ensure that a conformational change of the HsCen3 domain in MagFRET-1 was translated to a maximal change in relative orientation of the fluorescent domains, the final 8 amino acids from the flexible C-terminus of Cerulean were removed.

### *In vitro* characterization of MagFRET-1

**Figure 2:**
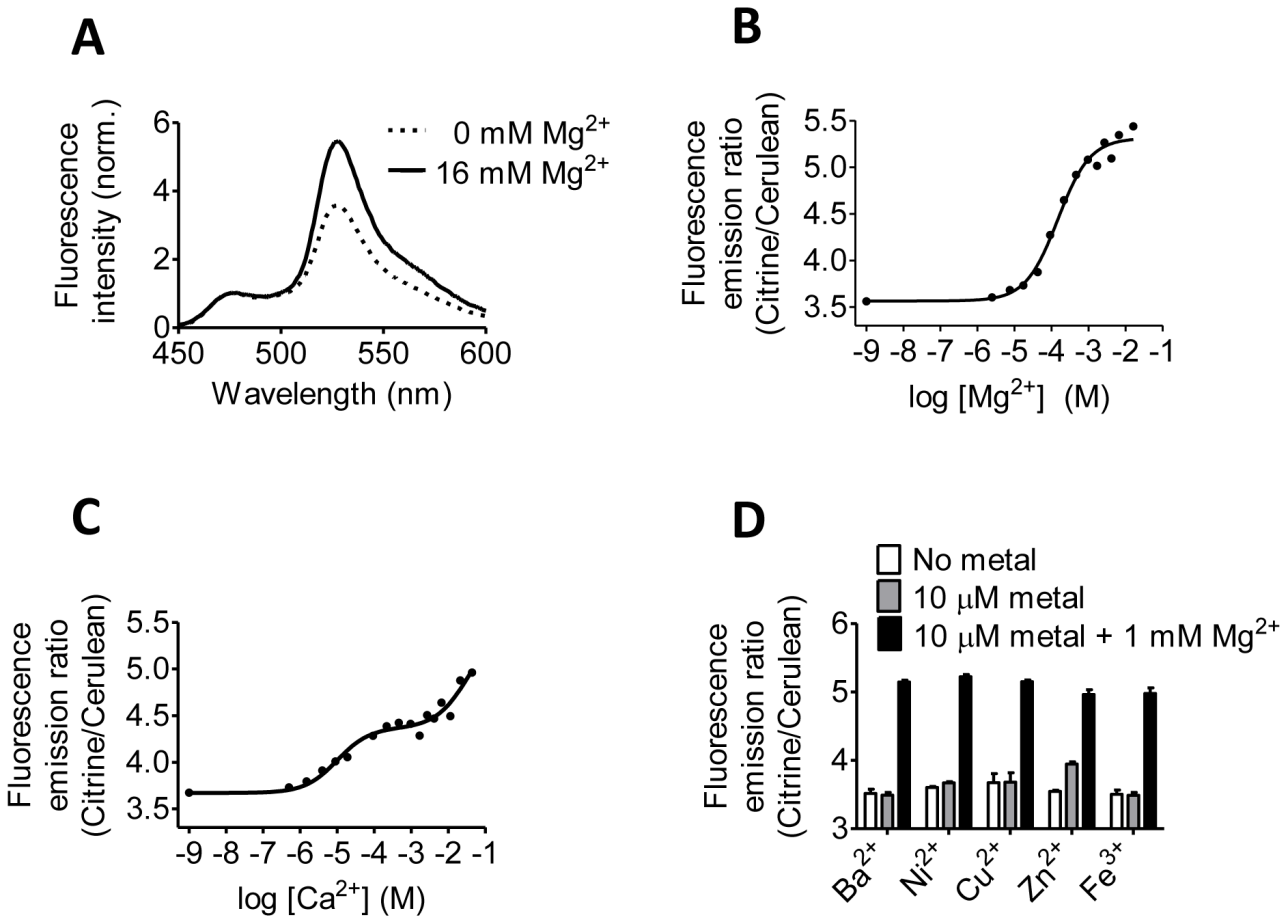
Metal binding properties of MagFRET-1. (A) Normalized fluorescence emission spectra of MagFRET-1 at 0 and at 16 mM Mg^2+^ after excitation at 420 nm. (B, C) Emission ratio (Citrine to Cerulean) of MagFRET-1 as a function of the Mg^2+^ (B) or Ca^2+^ (C) concentration. Solid lines indicate a fit to a single (B) or a double (C) binding event, yielding a *K*_d_ of 0.15±0.02 mM for Mg^2+^ and *K*_d_'s of 10±4 µM and ∼35 mM for Ca^2+^, respectively. (D) Emission ratios of MagFRET-1 in absence of metal, in the presence of 10 µM Ba^2+^, Ni^2+^, Cu^2+^, Zn^2+^ or Fe^3+^, and in the presence of the same metals and 1 mM Mg^2+^. Measurements were performed in triplicate, error bars indicate SEM. All measurements were performed in 150 mM Hepes (pH 7.1), 100 mM NaCl and 10% (v/v) glycerol with 0.2 µM sensor protein.

To allow characterization of MagFRET-1 *in vitro*, a His-tagged sensor construct was expressed in good yield in *E. coli* BL21(DE3) and purified using Ni^2+^-affinity and size exclusion chromatography. A relatively high ratio of Citrine to Cerulean emission of 3.6 was observed in the absence of Mg^2+^, indicating that the molten globule state of the metal binding domain is relatively compact bringing the fluorescent domains close together (**Figure 2A**). As expected, a further increase in emission ratio of 50% was observed upon addition of Mg^2+^, which is consistent with the formation of a more compact metal-bound, native state. The increase in emission ratio could be fitted using a 1∶1 binding model, yielding a *K*_d_ for Mg^2+^ of 148±23 µM (**Figure 2B**). Fortunately, this affinity is in the (lower) range of the cytosolic [Mg^2+^]_free_ reported by previous methods, and 10-fold weaker than that reported by Cox *et al.* for their N-terminal variant of HsCen3 [42]. Since HsCen3 was reported to not only bind Mg^2+^ but also contain two Ca^2+^ binding sites [42], the Ca^2+^ response of MagFRET-1 was also tested. Addition of Ca^2+^ led to a biphasic increase in emission ratio, which was fitted to a 2∶1 binding model (**Figure 2C**). Binding of Ca^2+^ to the high affinity site showed a *K*_d_ of 10±4 µM and resulted in a 19% increase in emission ratio. An additional 20% increase in emission ratio was observed at Ca^2+^ concentrations above 1 mM, but the low affinity for this site precluded accurate determination of its *K*_d_. While the absolute affinity of MagFRET-1 for Ca^2+^ is higher than for Mg^2+^, the sensor would not be expected to be sensitive to normal fluctuations in bulk cytosolic Ca^2+^ concentrations, which range between 0.1 and 1 µM [45]. No increase in emission ratio was observed upon addition of 10 µM Ba^2+^, Ni^2+^, Cu^2+^ or Fe^3+^, while only a very small increase was seen for 10 µM Zn^2+^ (**Figure 2D**), a concentration that is 10,000-fold higher than the free Zn^2+^ concentration found in the cytosol [36]. Another important aspect of sensor performance is pH sensitivity. Ca^2+^ and Mg^2+^ titrations performed at pH 6 and pH 8 showed that metal binding affinities were unaffected within this pH range (**Figure S4A–D**). As expected, the absolute emission ratios where somewhat lower at pH 6, due to the pH sensitivity of Citrine, which has a pK_a_ of 5.7 [44]. Finally, we noticed that the emission ratio of the apo form of the sensor is dependent on the ionic strength of the buffer (**Figure S5A**). When the Mg^2+^ titration was repeated in a low ionic strength buffer, the emission ratio of the apo form decreased to 2.2, whereas the emission ratio in the Mg^2+^-bound state was the same (**Figure S5B**) and the Mg^2+^ affinity remained mostly unaffected (*K*_d_ = 231±10 µM). This ionic strength dependence most likely reflects the influence of ionic strength on the compactness of the molten globule structure of HsCen3. Although the effect is less pronounced at physiologically relevant salt concentrations, it does mean that large changes in ionic strength should be avoided when applying MagFRET-1 *in situ*.

### Tuning metal binding affinities

**Figure 3:**
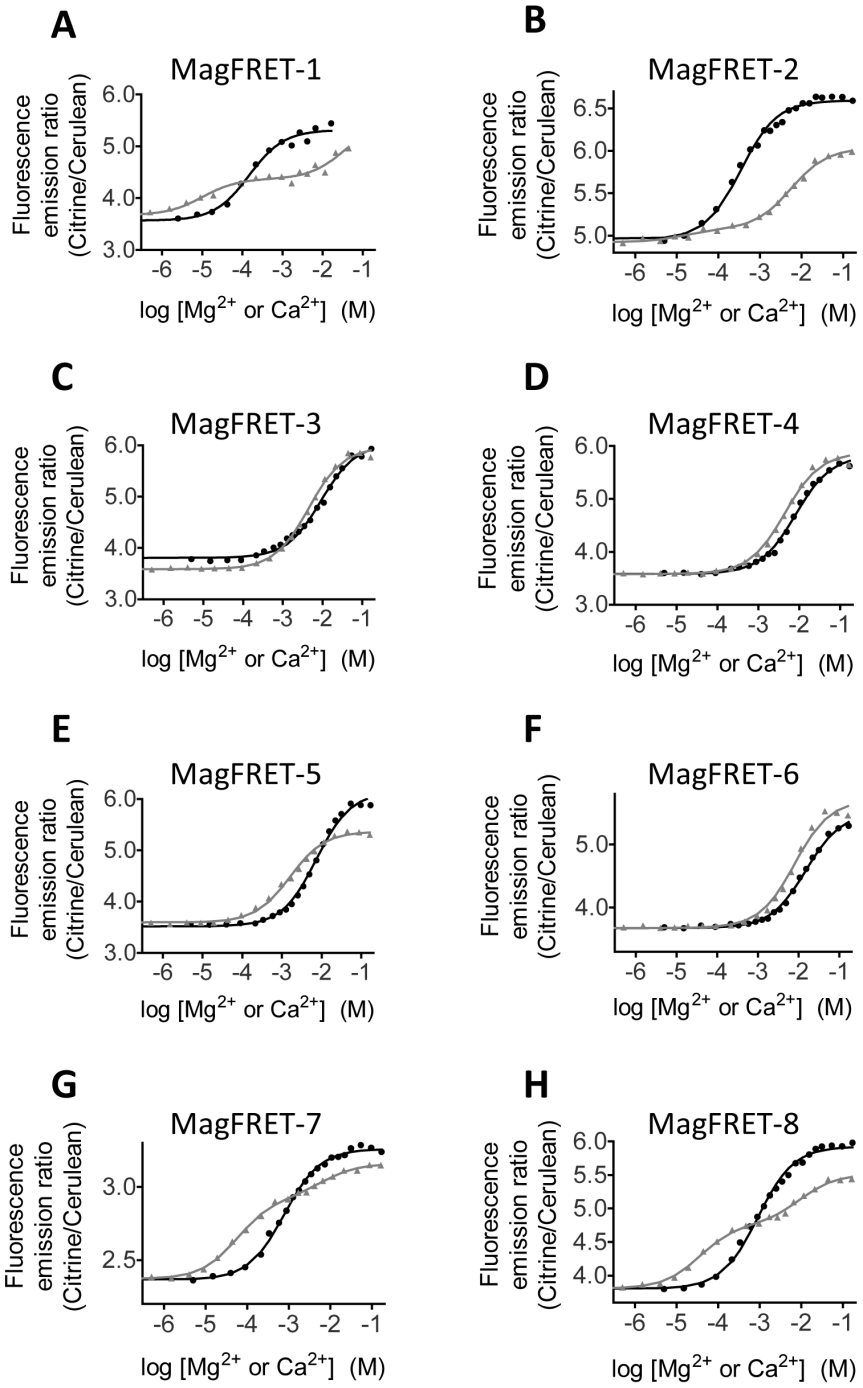
Mg^2+^/Ca^2+^ titrations of MagFRET variants with mutations in metal binding sites. (A–H) Emission ratio (Citrine to Cerulean) of MagFRET variants as a function of the Mg^2+^ (black circles) or Ca^2+^ (grey triangles) concentration. Solid black traces indicate a fit to a Mg^2+^-binding event, while grey traces indicate a fit to either double Ca^2+^-binding events (A, B, G, H) or a single Ca^2+^-binding event (C–F). Results of the titrations are summarized in Table 1. Measurements were performed in 150 mM Hepes (pH 7.1), 100 mM NaCl and 10% (v/v) glycerol and 0.2 (A) or 1 (B–H) µM protein.

To test whether we could further tune the metal affinity and specificity of the MagFRET sensor we explored several mutations in both metal binding sites. Targeting key residues in the 1^st^ EF hand (D1A, D3E, A7D and D5E/A7E) resulted in a reduction of both the Mg^2+^ and Ca^2+^ affinity to the millimolar regime, indicating that these residues are indeed directly involved in high affinity metal binding (**Table 1**, **Figure 3C–F**). Only a single Ca^2+^ binding event was observed for these mutants, suggesting that the two EF hands in MagFRET-3-6 have a similar Ca^2+^ affinity, making the two binding events indistinguishable. Interestingly, an E6D substitution (MagFRET-2) did not alter the affinity for Mg^2+^ or Ca^2+^ (**Figure 3B** **,** **Table 1**), showing that the presence of a glutamic acid at this position is not essential for high affinity metal binding. Although the change in emission ratio for binding Mg^2+^ is attenuated to 33% in this variant, the response to Ca^2+^ binding is almost absent for the high affinity site (3%), rendering this variant effectively Ca^2+^ insensitive. In an effort to abolish Ca^2+^ binding to the weakly Ca^2+^-binding EF hand II, we replaced aspartic acid 1 (MagFRET-7) and glycine 6 (MagFRET-8) at that site by positively charged lysine residues. Surprisingly, upon titration of Ca^2+^, both sensor variants still displayed the same biphasic response as seen with MagFRET-1 (**Figure 3A, G, H**), showing that neither of these residues is essential for the low affinity Ca^2+^ binding event in EF hand II. Interestingly, both the D1K and the G6K mutation subtly attenuated the high affinity mixed Ca^2+^/Mg^2+^ site in EF-hand I, leading to a 6- and 5-fold decrease of the Mg^2+^ affinity and a 6- and 4-fold decrease in Ca^2+^ affinity, respectively (**Figure 3G, H**). The somewhat weaker affinities for both Mg^2+^ and Ca^2+^ observed for MagFRET-7 and MagFRET-8 could prove beneficial for imaging Mg^2+^ homeostasis under conditions where the intracellular Mg^2+^ concentrations are higher.

**Table 1:** Sensor properties of the different MagFRET variants.

Variant	1^st^ EF-h and sequence^1^	2^nd^ EF-hand sequence^1^	*K*_d_ Mg^2+^/mM ± SE^2^	D.R. Mg^2+^ binding event^3^	*K*_d,1_ Ca^2+^/µM ± SE^2^	D.R. 1^st^ Ca^2+^ binding event^3^
MagFRET-1	DTDKDEAIDYHE	DREATGKITFED	0.15±0.02	49%	10±3.7	19%
MagFRET-2	DTDKD**D**AIDYHE	DREATGKITFED	0.35±0.03	33%	15±9.8	3.1%
MagFRET-3	**A**TDKDEAIDYHE	DREATGKITFED	9.2±0.7	58%	4500±243	66%
MagFRET-4	DT**E**KDEAIDYHE	DREATGKITFED	8.5±0.5	62%	4500±311	64%
MagFRET-5	DTDKDE**D**IDYHE	DREATGKITFED	7.4±0.5	74%	1600±116	49%
MagFRET-6	DTDK**E**E**E**IDYHE	DREATGKITFED	15±0.8	50%	7900±786	55%
MagFRET-7	DTDKDEAIDYHE	**K**REATGKITFED	0.78±0.04	38%	57±5	23%
MagFRET-8	DTDKDEAIDYHE	DREAT**K**KITFED	0.89±0.06	56%	36±5	25%

1 Mutations introduced in the first or second 12-residue metal binding loops of HsCen3 are indicated in bold and are underlined.

2 The dissociation constant (*K*_d_) for each variant's Mg^2+^ and first Ca^2+^ binding event is indicated, together with the standard error (SE).

3 A binding event's dynamic range (D.R.) is defined as the difference in emission ratio between the unbound and fully metal bound form divided by the emission ratio in the unbound form, multiplied by 100%.

### *In situ* characterization of MagFRET-1 in HEK293 cells

**Figure 4:**
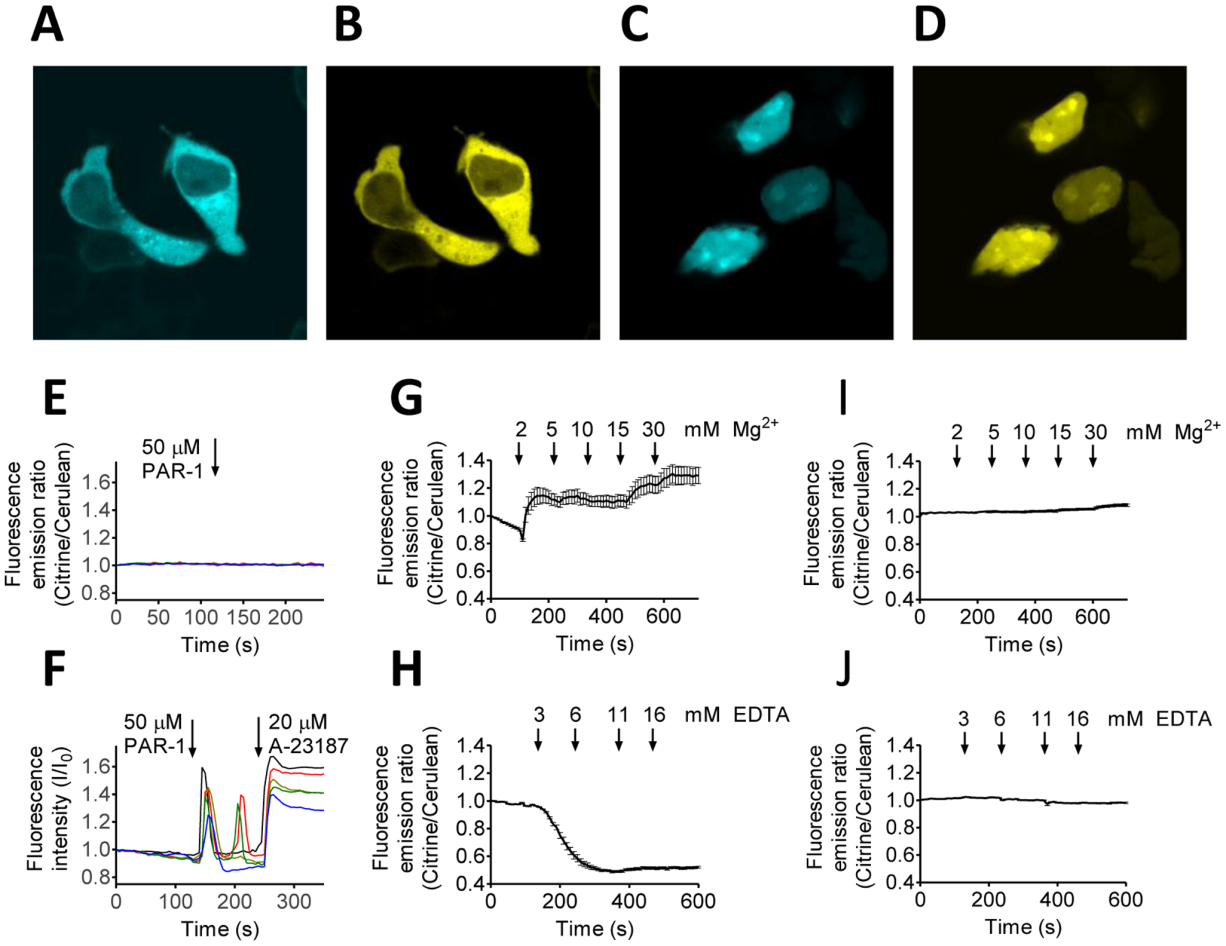
*In situ* characterization of MagFRET-1 in HEK293 cells. (A–D) Confocal fluorescence microscopy images showing HEK293 cells expressing MagFRET-1 (A, B) and MagFRET-1-NLS (C, D) showing Cerulean (A,C) or Citrine emission (B, D). (E, F) Investigation of MagFRET-1's *in situ* Ca^2+^ sensitivity. (E) Emission ratio over time of intact HEK293 cells expressing MagFRET-1 measured by widefield fluorescence microscopy. At t = 120 s, 50 µM of PAR-1 agonist peptide was added to activate Ca^2+^ signaling. (F) To confirm Ca^2+^ signaling took place in stimulated cells, the fluorescence intensity of intact HEK293 cells loaded with Ca^2+^-dye Oregon Green–BAPTA was followed. At t = 120 s, 50 µM of PAR-1 agonist peptide was added to activate Ca^2+^ signaling, and at t = 240 s, 20 µM A23187 was added. In E and F, each trace represents the response of an individual cell, with ratio (E) or intensity (F) normalized to the value at t = 0 s. (G, H) Response of MagFRET-1 expressed in permeabilized HEK293 cells to changes in [Mg^2+^]. MagFRET-1 emission ratio was followed over time as the concentration of MgCl_2_ (G) or EDTA (H) was increased, as indicated on the panels. (I, J) Response of negative control construct Cerulean-linker-Citrine expressed in permeabilized HEK293 cells to changes in [Mg^2+^]. To maintain an isotonic solution, the increase in Cl^−^ concentration due to addition of MgCl_2_ was compensated for by reducing the KCl concentration in the buffer. Prior to imaging, cells were permeabilized using 10 µg/mL digitonin. Traces in G to J represent averages of at least 9 cells, error bars indicate SEM, ratios were normalized to the emission ratio at t = 0.

To assess the sensor properties of MagFRET-1 *in situ*, CMV vectors were constructed to allow transient expression of MagFRET-1 in HEK293 cells. Fluorescence microscopy images revealed homogeneous expression of the sensor in the cytosol (**Figure 4A, B**) and Western blot analysis showed a single band corresponding to the full-length protein (**Figure S6**). In addition, transfection of cells with a construct containing three repeats of the nuclear localization sequence PKKKRKV [46], [47] at the C-terminus of the sensor protein (MagFRET-1-NLS), resulted in a clear cyan and yellow emission in the nucleus (**Figure 4C, D**), which demonstrates the ability to target MagFRET-1 to a specific location in the cell. Although the *K*_d_ of MagFRET-1 for Ca^2+^ determined *in vitro* is an order of magnitude higher than the maximum Ca^2+^ concentration that is typically observed in the cytosol during signaling, it was still important to verify that the MagFRET-1 sensor does not respond to stimuli that are known to transiently induce increases in cytosolic Ca^2+^ concentrations. Ca^2+^ signaling in HEK293 cells was activated via addition of 50 µM of the protease activated receptor-1 (PAR-1) agonist peptide [48], [49]. No changes in the emission ratio of MagFRET-1 were observed after addition of this stimulant (**Figure 4E**). Cells loaded with the synthetic Ca^2+^ dye Oregon Green-BAPTA did show a transient increase in fluorescence upon addition of PAR-1 agonist peptide, confirming that the expected increase in intracellular Ca^2+^ concentration was induced under these conditions (**Figure 4F**). Similar results were obtained with ATP, another commonly used stimulant for Ca^2+^ signaling [50]–[52]. Addition of 50 µM ATP did not affect the emission ratio of MagFRET-1 in HEK293 cells (**Figure S7A**), while cells loaded with Oregon Green-BAPTA showed a clear response to the same treatment (**Figure S7B**).

Having established that MagFRET-1 could be targeted and that it was insensitive to normal cytosolic Ca^2+^ fluctuations, we next characterized MagFRET-1's ability to report on changes in cytosolic [Mg^2+^]_free_. Surprisingly, attempts to perturb the free concentration of Mg^2+^ in the cytosol of intact HEK293 cells using previously reported procedures did not induce a significant ratiometric response in HEK293 cells transfected with MagFRET-1 (not shown). These protocols included incubation of the cells in 50 mM MgCl_2_ [29], exposure to the Mg^2+^ competitor Li^+^ [53] and varying of the sodium concentration to affect the Mg^2+^/Na^+^ exchanger [54]. To verify that the transiently expressed sensor is still responsive, we permeabilized HEK293 cells transfected with MagFRET-1 using 10 µg/mL digitonin and exposed them to buffers with different Mg^2+^ or EDTA concentrations (**Figure 4G–J**). Addition of 2 mM Mg^2+^ resulted in an increase in emission ratio for MagFRET-1, indicating metal binding to this sensor (**Figure 4G**). Subsequent addition of increasing Mg^2+^ concentrations did not result in a further increase in emission ratio up to 10 mM, while rounding of cells was observed at concentrations of 15 and 30 mM Mg^2+^ (not shown). To verify that the sensor could also monitor a decrease in cytosolic Mg^2+^ levels, the metal chelator EDTA was added to permeabilized cells expressing MagFRET-1. Upon addition of EDTA, cells expressing MagFRET-1 showed a decrease in emission ratio that is consistent with a decrease in cytosolic Mg^2+^ levels (**Figure 4H**). Importantly, no changes in emission ratio were observed upon addition of Mg^2+^ or EDTA to digitonin-treated cells expressing a negative control construct consisting of Cerulean, a flexible linker and Citrine but lacking any metal binding sites (**Figure 4I, J**). These results exclude the possibility that changes in emission ratio observed in MagFRET-1-expressing cells may have resulted from changes in the fluorescent domains' properties due to their sensitivity to pH or [Cl^−^] and confirm that MagFRET-1 is capable of responding to changes in intracellular Mg^2+^ levels.

## Discussion

To the best of our knowledge, this work represents the first report of a genetically encoded fluorescent sensor for Mg^2+^. The new sensor principle of metal-induced folding of an EF-hand protein was used to create a FRET-based sensor protein that combines a physiologically relevant Mg^2+^ affinity with a 50% increase in emission ratio upon Mg^2+^ binding. Mutations introduced in the metal binding domains yielded sensor variants with different degrees of attenuation in Mg^2+^ affinity, generating a toolbox of MagFRET variants for different applications. Unlike most synthetic fluorescent Mg^2+^ probes reported so far, MagFRET-1 allows emission ratiometric detection of Mg^2+^ and is thus less sensitive to fluctuations in sensor concentration or background fluorescence. A general advantage of genetically encoded sensors is that their subcellular localization can be easily controlled, as we demonstrated by targeting MagFRET-1 to the cytosol and nucleus of HEK293 cells. Importantly, while MagFRET-1 is also sensitive to Ca^2+^, its Ca^2+^ affinity is sufficiently attenuated to make the sensor effectively unresponsive to the Ca^2+^ levels reached during signaling. Although MagFRET-1 was clearly responsive to changes in Mg^2+^ in permeabilized HEK293 cells, we did not observe similar changes in emission ratio for intact cells. A lack of selective Mg^2+^ ionophores and chelators is a fundamental problem in the Mg^2+^ imaging field [4] and prevented us from calibrating the sensor's resting emission ratio by depleting and saturating the sensor *in situ*, as is commonly done for genetically encoded Ca^2+^ and Zn^2+^ sensors.

Despite the intrinsically large conformational change associated with protein folding, surprisingly few examples of FRET sensors exist where ligand-induced folding of a partially unfolded receptor domain is employed in FRET sensor design. Most FRET sensors developed so far either use ligand binding domains that are known to undergo significant conformational changes upon ligand binding (e.g. the periplasmic binding proteins [55]), or receptor domains that undergo a ligand-induced interaction with another peptide/protein domain (e.g. Cameleons [32]). Ligand-induced folding is believed to occur for intrinsically-disordered proteins, which may account for 35–51% of all eukaryotic protein domains [56]. In addition, ligand binding domains can be intentionally destabilized to turn conformationally silent ligand binding domains into attractive input domains for FRET sensor design [57]. This work revealed that ligand-induced folding of intrinsically-disordered proteins is an attractive mechanism for FRET sensor design. However, it also identified a potential disadvantage as we observed that the conformation and thus the amount of energy transfer of the ligand-free state is sensitive to ionic strength. Although this effect is most apparent below physiologically relevant salt concentrations, it is important to be aware of this phenomenon and use these sensors under conditions of constant ionic strength or use appropriate control sensors that have a strongly attenuated Mg^2+^ affinity, such as e.g. MagFRET-6.

The Mg^2+^ and Ca^2+^ affinity of MagFRET-1 were found to be attenuated compared to the affinities previously reported for the N-terminal domain of HsCen3 (*K*_d_ = 10–28 µM for Mg^2+^; *K*_d_ = 1.5–8 and 140 µM for Ca^2+^). The difference in metal binding affinity might be explained by the fact that the central α-helix that connects the 2^nd^ and 3^rd^ EF hands in HsCen3 was reduced to half its length in MagFRET-1, possibly further destabilizing the N-terminal domain, resulting in a net decrease in Mg^2+^ affinity. Fortunately, in this case the attenuation yielded a sensor that is sensitive to physiologically relevant Mg^2+^ concentrations, and insensitive to normal cytosolic Ca^2+^ concentrations. The limited number of metal binding domain mutations that were explored in this study revealed that mutations in the first EF hand typically result in strongly attenuated metal binding affinities. A more subtle, 4–6 fold attenuation of metal binding affinity was obtained after introduction of positively charged amino acids in the 2^nd^ EF hand. These effects could be due to direct allosteric coupling between the two EF hands in the metal-bound state, but alternatively could also result from further stabilization of the molten globule state. An important goal is to develop sensor variants that are less sensitive to Ca^2+^ yet retain affinity for Mg^2+^, as this would allow targeting to organelles known to have much higher resting levels of Ca^2+^. The similar coordination chemistries of Ca^2+^ and Mg^2+^ make rational design of such variants challenging, although mutations in an EF-hand-like protein have been reported that decreased the Ca^2+^ affinity 100-fold while simultaneously doubling the Mg^2+^ affinity [58]. Further optimization of metal binding affinity and specificity, but also the sensor's dynamic range, may benefit from directed evolution approaches similar to the ones that were recently applied to develop new color variants of the Ca^2+^ sensor GECO [34], [59].

*In situ* characterization of MagFRET-1 in HEK293 cells revealed that the sensor is readily expressed in the cytosol and can be targeted to the nucleus. Two ligands that are known to induce Ca^2+^ signaling in cells, PAR-1 agonist peptide and ATP, did not affect the emission ratio of the MagFRET-1 sensor in HEK 293 cells. This result was expected based on the affinity of MagFRET-1 that was determined *in vitro* (*K*_d_ = 10 µM) and previous reports that show that bulk cytosolic Ca^2+^ concentrations typically reach a maximum of 1 µM during signaling [45]. In contrast, synthetic Mg^2+^ dyes with similar affinity to Ca^2+^ as MagFRET-1 have been reported to respond to Ca^2+^. This may be explained by the fact that MagFRET-1 is exclusively localized in the cytosol, whereas synthetic dyes sometimes partially mislocalize to Ca^2+^-rich organelles such as the ER or even leak into the external buffer [60]. Surprisingly, HEK293 cells expressing MagFRET-1 did not respond to procedures that were previously reported to affect the intracellular Mg^2+^ concentration. A possible explanation is that the free concentration of Mg^2+^ in the cytosol is tightly buffered and controlled and not easily changed by external stimuli. The free concentration of Mg^2+^ in the cytosol is at least 1000-fold higher than that of Ca^2+^ and 10^6^-fold higher than that of Zn^2+^. For this reason and because Mg^2+^ is essential to such a wide variety of biological processes, it would not be surprising that manipulation of intracellular free Mg^2+^ is much more difficult than that of other metals. Nonetheless, it is conceivable that while the overall free Mg^2+^ concentration in cells is relatively constant, substantial and physiologically relevant fluctuations in Mg^2+^ concentration could still occur locally, e.g. at the plasma membrane near Mg^2+^-specific ion channels. Although MagFRET-1 responded to changes in Mg^2+^ concentration in the order of seconds both *in vitro* and in cells, protein-based sensors often display slower kinetics than small molecule sensors, so that MagFRET-1 might fail to respond to extremely fast Mg^2+^ transients, should they occur. In addition, overall Mg^2+^ levels could change over longer periods of time, e.g. as a function of the cell cycle. These changes may be more reliably monitored using lifetime imaging, which might also be the preferred method to allow quantification of intracellular Mg^2+^ concentrations. Although we confirmed that MagFRET-1 is responsive to changes in Mg^2+^ concentration in permeabilized cells, we cannot completely rule out that for some unknown reason MagFRET-1 is less responsive in intact cells. The potential and limitations of the MagFRET sensors for intracellular Mg^2+^ imaging therefore remain to be further established.

## Supporting Information

Figure S1 **Nucleotide sequence of bacterial expression vector pET28a-MagFRET-1 ORF.** The DNA sequence is shown in lowercase, with the single letter amino acid code shown beneath each codon in uppercase. The His-tag is highlighted in bright green, the thrombin cleavage site in pink, Cerulean in turquoise, HsCen3 in red and Citrine in yellow. The two EF-hand motifs are underlined in white.
(PDF)

Figure S2 **Nucleotide sequence of mammalian expression vector pCMV-MagFRET-1 ORF.** The DNA sequence is shown in lowercase, with the single letter amino acid code shown beneath each codon in uppercase. Cerulean is highlighted in turquoise, HsCen3 in red and Citrine in yellow. The two EF-hand motifs are underlined in white.
(PDF)

Figure S3 **Nucleotide sequence of mammalian expression vector pCMV-MagFRET-1-NLS ORF.** The DNA sequence is shown in lowercase, with the single letter amino acid code shown beneath each codon in uppercase. Cerulean is highlighted in turquoise, HsCen3 in red, Citrine in yellow and the three PKKKRKV repeats in grey. The two EF-hand motifs are underlined in white.
(PDF)

Figure S4 **Effect of pH on MagFRET-1.** To check for pH sensitivity, the MagFRET-1 emission ratio was followed as a function of Mg^2+^ (A, B) and Ca^2+^ (C, D) concentration, at pH 6 (A, C) and pH 8 (B, D). Fitting of the data revealed a MagFRET-1 *K*_d_ for Mg^2+^ of 230±35 µM at pH 6 and 99±18 µM at pH 8. The sensor's *K*_d_ for Ca^2+^ (first binding event) at pH = 6 was found to be 5.6±1.7 µM, while at pH 8 it was 5.9±1.9 µM. Buffers used were 150 mM MES (pH 6), 100 mM NaCl and 10% glycerol for pH 6 and 150 mM Tris (pH 8), 100 mM NaCl and 10% glycerol for pH 8.
(TIF)

Figure S5 **Effect of ionic strength on MagFRET-1.** (A) Emission ratio of MagFRET-1 at increasing concentrations of ammonium acetate or NaCl in a buffer with low ionic strength. (B) Emission ratio of MagFRET-1 as a function of Mg^2+^ concentration in a buffer with low ionic strength. The low ionic strength buffer used in (A, B) was 20 mM Hepes (pH 7.1), 10 mM NaCl, 10% (v/v) glycerol. Fitting of the data using a single binding event revealed a *K*_d_ for Mg^2+^ of 231±10 µM.
(TIF)

Figure S6 **Western blot analysis of MagFRET-1 expressing HEK293 cells.** A molecular weight marker (Precision Plus Protein Standards, Bio-Rad) was loaded in the left-hand lane. Lane 1 displays the lysate of HEK293 cells transfected with a vector encoding for MagFRET-1 under control of a CMV promoter. The blotting membrane was incubated with mouse anti-GFP (Ab3277, Abcam), followed by HRP-functionalized goat anti-mouse antibody (Dako). The calculated molecular weight for MagFRET-1 is 62 kDa.
(TIF)

Figure S7 **Investigation of MagFRET-1 response to elevated cytosolic Ca^2+^ induced by ATP.** (A) Emission ratio over time of intact HEK293 cells expressing MagFRET-1 measured by widefield fluorescence microscopy. At t = 104 s, 50 µM ATP was added to activate Ca^2+^ signaling. (B) To confirm Ca^2+^ signaling took place in stimulated cells, the fluorescence intensity of intact HEK293 cells loaded with Ca^2+^-dye Oregon Green–BAPTA was followed. At t = 104 s, 50 µM ATP was added to activate Ca^2+^ signaling, and at t = 226 s, 20 µM of the Ca^2+^ ionophore A23187 was added. In A and B, each trace represents the response of an individual cell, with ratio (A) or intensity (B) normalized to the value at t = 0 s.
(TIF)

Method S1 **Western blotting.**
(PDF)

Table S1 **Primers used for mutagenesis of HsCen3.**
(PDF)

## References

[1] Shi J, Krishnamoorthy G, Yang Y, Hu L, Chaturvedi N, et al (2002) Mechanism of magnesium activation of calcium-activated potassium channels. Nature 418: 876–880. 12192410 10.1038/nature00941

[2] Nadler MJ, Hermosura MC, Inabe K, Perraud AL, Zhu Q, et al (2001) LTRPC7 is a Mg.ATP-regulated divalent cation channel required for cell viability. Nature 411: 590–595. 11385574 10.1038/35079092

[3] Romani AM (2007) Magnesium homeostasis in mammalian cells. Front Biosci 12: 308–331. 17127301 10.2741/2066

[4] Grubbs RD (2002) Intracellular magnesium and magnesium buffering. Biometals 15: 251–259. 12206391 10.1023/a:1016026831789

[5] Rutter GA, Osbaldeston NJ, McCormack JG, Denton RM (1990) Measurement of matrix free Mg^2+^ concentration in rat heart mitochondria by using entrapped fluorescent probes. Biochem J 271: 627–634. 2244870 10.1042/bj2710627 PMC1149608

[6] Gunther T (2006) Concentration, compartmentation and metabolic function of intracellular free Mg^2+^ . Magnes Res 19: 225–236. 17402290

[7] Corkey BE, Duszynski J, Rich TL, Matschinsky B, Williamson JR (1986) Regulation of free and bound magnesium in rat hepatocytes and isolated mitochondria. J Biol Chem 261: 2567–2574. 3081495

[8] Maguire ME (2006) The structure of CorA: a Mg^2+^-selective channel. Curr Opin Struct Biol 16: 432–438. 16828282 10.1016/j.sbi.2006.06.006

[9] Schlingmann KP, Waldegger S, Konrad M, Chubanov V, Gudermann T (2007) TRPM6 and TRPM7-Gatekeepers of human magnesium metabolism. Biochim Biophys Acta, Molecular Basis of Disease 1772: 813–821. 10.1016/j.bbadis.2007.03.009 17481860

[10] Hattori M, Tanaka Y, Fukai S, Ishitani R, Nureki O (2007) Crystal structure of the MgtE Mg^2+^ transporter. Nature 448: 1072–1075. 17700703 10.1038/nature06093

[11] Konrad M, Weber S (2003) Recent advances in molecular genetics of hereditary magnesium-losing disorders. J Am Soc Nephrol 14: 249–260. 12506158 10.1097/01.asn.0000049161.60740.ce

[12] Killilea DW, Ames BN (2008) Magnesium deficiency accelerates cellular senescence in cultured human fibroblasts. Proc Natl Acad Sci U S A 105: 5768–5773. 18391207 10.1073/pnas.0712401105 PMC2311331

[13] Chaudhary DP, Sharma R, Bansal DD (2010) Implications of magnesium deficiency in type 2 diabetes: a review. Biol Trace Elem Res 134: 119–129. 19629403 10.1007/s12011-009-8465-z

[14] Bo S, Pisu E (2008) Role of dietary magnesium in cardiovascular disease prevention, insulin sensitivity and diabetes. Curr Opin Lipidol 19: 50–56. 18196987 10.1097/MOL.0b013e3282f33ccc

[15] Rude RK, Gruber HE (2004) Magnesium deficiency and osteoporosis: animal and human observations. J Nutr Biochem 15: 710–716. 15607643 10.1016/j.jnutbio.2004.08.001

[16] Li FY, Chaigne-Delalande B, Kanellopoulou C, Davis JC, Matthews HF, et al (2011) Second messenger role for Mg^2+^ revealed by human T-cell immunodeficiency. Nature 475: 471–476. 21796205 10.1038/nature10246 PMC3159560

[17] Trapani V, Farruggia G, Marraccini C, Iotti S, Cittadini A, et al (2010) Intracellular magnesium detection: imaging a brighter future. Analyst 135: 1855–1866. 20544083 10.1039/c0an00087f

[18] Cohen SM, Burt CT (1977) ^31^P nuclear magnetic relaxation studies of phosphocreatine in intact muscle: determination of intracellular free magnesium. Proc Natl Acad Sci U S A 74: 4271–4275. 270670 10.1073/pnas.74.10.4271 PMC431921

[19] Resnick LM, Gupta RK, Laragh JH (1984) Intracellular free magnesium in erythrocytes of essential hypertension: relation to blood pressure and serum divalent cations. Proc Natl Acad Sci U S A 81: 6511–6515. 6593713 10.1073/pnas.81.20.6511 PMC391954

[20] Gunther T (2007) Total and free Mg^2+^ contents in erythrocytes: a simple but still undisclosed cell model. Magnes Res 20: 161–167. 17972458

[21] Kim HM, Yang PR, Seo MS, Yi J-S, Hong JH, et al (2007) Magnesium ion selective two-photon fluorescent probe based on a benzo[h]chromene derivative for in vivo imaging. J Org Chem 72: 2088–2096. 17316048 10.1021/jo062341m

[22] Hwan Myung K, Cheol J, Bo Ra K, Soon-Young J, Jin Hee H, et al (2007) Environment-sensitive two-photon probe for intracellular free magnesium ions in live tissue. Angew Chem Int Ed Engl 46: 3460–3463. 17397120 10.1002/anie.200700169

[23] Komatsu H, Miki T, Citterio D, Kubota T, Shindo Y, et al (2005) Single molecular multianalyte (Ca^2+^, Mg^2+^) fluorescent probe and applications to bioimaging. J Am Chem Soc 127: 10798–10799. 16076163 10.1021/ja0528228

[24] Komatsu H, Iwasawa N, Citterio D, Suzuki Y, Kubota T, et al (2004) Design and synthesis of highly sensitive and selective fluorescein-derived magnesium fluorescent probes and application to intracellular 3D Mg^2+^ imaging. J Am Chem Soc 126: 16353–16360. 15600336 10.1021/ja049624l

[25] Raju B, Murphy E, Levy LA, Hall RD, London RE (1989) A fluorescent indicator for measuring cytosolic free magnesium. Am J Physiol Cell Physiol 256: C540–548. 10.1152/ajpcell.1989.256.3.C540 2923192

[26] Paredes RM, Etzler JC, Watts LT, Zheng W, Lechleiter JD (2008) Chemical calcium indicators. Methods 46: 143–151. 18929663 10.1016/j.ymeth.2008.09.025 PMC2666335

[27] Shindo Y, Fujii T, Komatsu H, Citterio D, Hotta K, et al (2011) Newly developed Mg^2+^-selective fluorescent probe enables visualization of Mg^2+^ dynamics in mitochondria. PLoS One 6: e23684. 21858208 10.1371/journal.pone.0023684 PMC3156752

[28] Hurley TW, Ryan MP, Brinck RW (1992) Changes of cytosolic Ca^2+^ interfere with measurements of cytosolic Mg^2+^ using mag-fura-2. Am J Physiol Cell Physiol 263: C300–307. 10.1152/ajpcell.1992.263.2.C300 1514577

[29] Zhou H, Clapham DE (2009) Mammalian MagT1 and TUSC3 are required for cellular magnesium uptake and vertebrate embryonic development. Proc Natl Acad Sci U S A 106: 15750–15755. 19717468 10.1073/pnas.0908332106 PMC2732712

[30] Xie J, Sun B, Du J, Yang W, Chen HC, et al (2011) Phosphatidylinositol 4,5-bisphosphate (PIP(2)) controls magnesium gatekeeper TRPM6 activity. Sci Rep 1: 146. 22180838 10.1038/srep00146 PMC3238349

[31] Palmer AE, Qin Y, Park JG, McCombs JE (2011) Design and application of genetically encoded biosensors. Trends Biotechnol 29: 144–152. 21251723 10.1016/j.tibtech.2010.12.004 PMC3433949

[32] Miyawaki A, Llopis J, Heim R, Michael McCaffery J, Adams JA, et al (1997) Fluorescent indicators for Ca^2+^ based on green fluorescent proteins and calmodulin. Nature 388: 882–887. 9278050 10.1038/42264

[33] Palmer AE, Giacomello M, Kortemme T, Hires SA, Lev-Ram V, et al (2006) Ca^2+^ indicators based on computationally redesigned calmodulin-peptide pairs. Chem Biol 13: 521–530. 16720273 10.1016/j.chembiol.2006.03.007

[34] Zhao YX, Araki S, Jiahui WH, Teramoto T, Chang YF, et al (2011) An expanded palette of genetically encoded Ca^2+^ indicators. Science 333: 1888–1891. 21903779 10.1126/science.1208592 PMC3560286

[35] Tian L, Hires SA, Mao T, Huber D, Chiappe ME, et al (2009) Imaging neural activity in worms, flies and mice with improved GCaMP calcium indicators. Nat Methods 6: 875–U113. 19898485 10.1038/nmeth.1398 PMC2858873

[36] Vinkenborg JL, Nicolson TJ, Bellomo EA, Koay MS, Rutter GA, et al (2009) Genetically encoded FRET sensors to monitor intracellular Zn^2+^ homeostasis. Nat Methods 6: 737–740. 19718032 10.1038/nmeth.1368 PMC6101214

[37] Dittmer PJ, Miranda JG, Gorski JA, Palmer AE (2009) Genetically encoded sensors to elucidate spatial distribution of cellular zinc. J Biol Chem 284: 16289–16297. 19363034 10.1074/jbc.M900501200 PMC2713558

[38] Miranda JG, Weaver AL, Qin Y, Park JG, Stoddard CI, et al (2012) New alternately colored FRET sensors for simultaneous monitoring of Zn^2+^ in multiple cellular locations. PLoS One 7: e49371. 23173058 10.1371/journal.pone.0049371 PMC3500285

[39] Qiao W, Mooney M, Bird AJ, Winge DR, Eide DJ (2006) Zinc binding to a regulatory zinc-sensing domain monitored in vivo by using FRET. Proc Natl Acad Sci U S A 103: 8674–8679. 16720702 10.1073/pnas.0600928103 PMC1482638

[40] Wegner SV, Arslan H, Sunbul M, Yin J, He C (2010) Dynamic copper(I) imaging in mammalian cells with a genetically encoded fluorescent copper(I) sensor. J Am Chem Soc 132: 2567–2569. 20131768 10.1021/ja9097324

[41] Koay MS, Janssen BM, Merkx M (2013) Tuning the metal binding site specificity of a fluorescent sensor protein: from copper to zinc and back. Dalton Trans 42: 3230–3232. 23076326 10.1039/c2dt32082g

[42] Cox JA, Tirone F, Durussel I, Firanescu C, Blouquit Y, et al (2005) Calcium and magnesium binding to human centrin 3 and interaction with target peptides. Biochemistry 44: 840–850. 15654740 10.1021/bi048294e

[43] Golynskiy MV, Rurup WF, Merkx M (2010) Antibody detection by using a FRET-based protein conformational switch. ChemBioChem 11: 2264–2267. 20928879 10.1002/cbic.201000143

[44] Griesbeck O, Baird GS, Campbell RE, Zacharias DA, Tsien RY (2001) Reducing the environmental sensitivity of yellow fluorescent protein. J Biol Chem 276: 29188–29194. 11387331 10.1074/jbc.M102815200

[45] Bootman MD (2012) Calcium signaling. Cold Spring Harb Perspect Biol 4: a011171. 22751152 10.1101/cshperspect.a011171 PMC3385957

[46] Kalderon D, Roberts BL, Richardson WD, Smith AE (1984) A short amino acid sequence able to specify nuclear location. Cell 39: 499–509. 6096007 10.1016/0092-8674(84)90457-4

[47] Fischer-Fantuzzi L, Vesco C (1988) Cell-dependent efficiency of reiterated nuclear signals in a mutant simian virus 40 oncoprotein targeted to the nucleus. Mol Cell Biol 8: 5495–5503. 2854199 10.1128/mcb.8.12.5495-5503.1988 PMC365653

[48] Jiang T, Danilo P Jr, Steinberg SF (1998) The thrombin receptor elevates intracellular calcium in adult rat ventricular myocytes. J Mol Cell Cardiol 30: 2193–2199. 9925357 10.1006/jmcc.1998.0779

[49] Hui KY, Jakubowski JA, Wyss VL, Angleton EL (1992) Minimal sequence requirement of thrombin receptor agonist peptide. Biochem Biophys Res Commun 184: 790–796. 1315534 10.1016/0006-291x(92)90659-9

[50] Isshiki M, Ando J, Korenaga R, Kogo H, Fujimoto T, et al (1998) Endothelial Ca^2+^ waves preferentially originate at specific loci in caveolin-rich cell edges. Proc Natl Acad Sci U S A 95: 5009–5014. 9560219 10.1073/pnas.95.9.5009 PMC20204

[51] Nagai T, Yamada S, Tominaga T, Ichikawa M, Miyawaki A (2004) Expanded dynamic range of fluorescent indicators for Ca^2+^ by circularly permuted yellow fluorescent proteins. Proc Natl Acad Sci U S A 101: 10554–10559. 15247428 10.1073/pnas.0400417101 PMC490022

[52] Qi Z, Murase K, Obata S, Sokabe M (2000) Extracellular ATP-dependent activation of plasma membrane Ca^2+^ pump in HEK-293 cells. Br J Pharmacol 131: 370–374. 10991933 10.1038/sj.bjp.0703563 PMC1572318

[53] Fonseca CP, Montezinho LP, Baltazar G, Layden B, Freitas DM, et al (2000) Li^+^ influx and binding, and Li^+^/Mg^2+^ competition in bovine chromaffin cell suspensions as studied by ^7^Li NMR and fluorescence spectroscopy. Met Based Drugs 7: 357–364. 18475968 10.1155/MBD.2000.357 PMC2365240

[54] Schweigel M, Park HS, Etschmann B, Martens H (2006) Characterization of the Na^+^-dependent Mg^2+^ transport in sheep ruminal epithelial cells. Am J Physiol Gastrointest Liver Physiol 290: G56–65. 16109844 10.1152/ajpgi.00014.2005

[55] Okumoto S, Looger LL, Micheva KD, Reimer RJ, Smith SJ, et al (2005) Detection of glutamate release from neurons by genetically encoded surface-displayed FRET nanosensors. Proc Natl Acad Sci U S A 102: 8740–8745. 15939876 10.1073/pnas.0503274102 PMC1143584

[56] Dunker AK, Brown CJ, Lawson JD, Iakoucheva LM, Obradovic Z (2002) Intrinsic disorder and protein function. Biochemistry 41: 6573–6582. 12022860 10.1021/bi012159+

[57] Kohn JE, Plaxco KW (2005) Engineering a signal transduction mechanism for protein-based biosensors. Proc Natl Acad Sci U S A 102: 10841–10845. 16046542 10.1073/pnas.0503055102 PMC1182433

[58] Wang W, Barnabei MS, Asp ML, Heinis FI, Arden E, et al (2013) Noncanonical EF-hand motif strategically delays Ca^2+^ buffering to enhance cardiac performance. Nat Med 19: 305–312. 23396207 10.1038/nm.3079 PMC3727912

[59] Lindenburg L, Merkx M (2012) Colorful calcium sensors. ChemBioChem 13: 349–351. 22234942 10.1002/cbic.201100739

[60] Trapani V, Schweigel-Rontgen M, Cittadini A, Wolf FI (2012) Intracellular magnesium detection by fluorescent indicators. Methods Enzymol 505: 421–444. 22289466 10.1016/B978-0-12-388448-0.00030-9

