# Supplementary material for: MagFRET: The First Genetically Encoded Fluorescent Mg2+ Sensor
Source: PLoS One. 2013 Dec 2;8(12):e82009. doi: 10.1371/journal.pone.0082009 (PMC3846734; doi:10.1371/journal.pone.0082009)
Supplement: Figure S1 — Nucleotide sequence of bacterial expression vector pET28a-MagFRET-1 ORF. The DNA sequence is shown in lowercase, with the single letter amino acid code shown beneath each codon in uppercase. The His-tag is highlighted in bright green, the thrombin cleavage site in pink, Cerulean in turquoise, HsCen3 in red and Citrine in yellow. The two EF-hand motifs are underlined in white. (PDF) [file pone.0082009.s001.pdf]

Figure S1: pET28a-MagFRET-1

```
   1  atgggcagcagccatcatcatcatcatcacagcagcggcctggtgccgcgcggcagccat   60
       M  G  S  S  H  H  H  H  H  H  S  S  G  L  V  P  R  G  S  H
  61  atggtgagcaagggcgaggagctgttcaccggggtggtgcccatcctggtcgagctggac  120
       M  V  S  K  G  E  E  L  F  T  G  V  V  P  I  L  V  E  L  D
 121  ggcgacgtaaacggccacaagttcagcgtgtccggcgagggcgagggcgatgccacctac  180
       G  D  V  N  G  H  K  F  S  V  S  G  E  G  E  G  D  A  T  Y
 181  ggcaagctgaccctgaagttcatctgcaccaccggtaagctgcccgtgccctggcccacc  240
       G  K  L  T  L  K  F  I  C  T  T  G  K  L  P  V  P  W  P  T
 241  ctcgtgaccaccctgacctggggcgtgcagtgcttcgcccgctaccccgaccacatgaag  300
       L  V  T  T  L  T  W  G  V  Q  C  F  A  R  Y  P  D  H  M  K
 301  cagcacgacttcttcaagtccgccatgcccgaaggctacgtccaggagcgcaccatcttc  360
       Q  H  D  F  F  K  S  A  M  P  E  G  Y  V  Q  E  R  T  I  F
 361  ttcaaggacgacggcaactacaagacccgcgccgaggtgaagttcgagggcgacaccctg  420
       F  K  D  D  G  N  Y  K  T  R  A  E  V  K  F  E  G  D  T  L
 421  gtgaaccgcatcgagctgaagggcatcgacttcaaggaggacggcaacatcctggggcac  480
       V  N  R  I  E  L  K  G  I  D  F  K  E  D  G  N  I  L  G  H
 481  aagctggagtacaacgccatcagcgacaacgtctatatcaccgccgacaagcagaagaac  540
       K  L  E  Y  N  A  I  S  D  N  V  Y  I  T  A  D  K  Q  K  N
 541  ggcatcaaggccaacttcaagatccgccacaacatcgaggacggcagcgtgcagctcgcc  600
       G  I  K  A  N  F  K  I  R  H  N  I  E  D  G  S  V  Q  L  A
 601  gaccactaccagcagaacacccccatcggcgacggccccgtgctgctgcccgacaaccac  660
       D  H  Y  Q  Q  N  T  P  I  G  D  G  P  V  L  L  P  D  N  H
 661  tacctgagcacccagtccgccctgagcaaagaccccaacgagaagcgcgatcacatggtc  720
       Y  L  S  T  Q  S  A  L  S  K  D  P  N  E  K  R  D  H  M  V
 721  ctgctggagttcgtgaccgccgccgggatcactagcgaggaacagaaacaagaaattaaa  780
       L  L  E  F  V  T  A  A  G  I  T  S  E  E  Q  K  Q  E  I  K
 781  gatgcttttgaactgtttgatacagacaaagatgaagcaatagattatcatgaactgaag  840
       D  A  F  E  L  F  D  T  D  K  D  E  A  I  D  Y  H  E  L  K
 841  gtggcaatgagagccttggggtttgatgtaaaaaaagctgatgtactgaagattcttaaa  900
       V  A  M  R  A  L  G  F  D  V  K  K  A  D  V  L  K  I  L  K
 901  gattatgacagagaagccacagggaaaatcacctttgaagatttaatgaagttgtgaca  960
       D  Y  D  R  E  A  T  G  K  I  T  F  E  D  F  N  E  V  V  T
 961  gactggatattggaaagagatgccatggtgagcaagggcgaggagctgttcaccggggtg  1020
       D  W  I  L  E  R  D  A  M  V  S  K  G  E  E  L  F  T  G  V
1021  gtgcccatcctggtcgagctggacggcgacgtaaacggccacaagttcagcgtgtccggc  1080
       V  P  I  L  V  E  L  D  G  D  V  N  G  H  K  F  S  V  S  G
1081  gagggcgagggcgatgccacctacggcaagctgaccctgaagttcatctgcaccaccggc  1140
       E  G  E  G  D  A  T  Y  G  K  L  T  L  K  F  I  C  T  T  G
1141  aagctgcccgtgccctggcccaccctcgtgaccaccttcggctacggcctgatgtgcttc  1200
       K  L  P  V  P  W  P  T  L  V  T  T  F  G  Y  G  L  M  C  F
1201  gcccgctaccccgaccacatgaagcagcacgacttcttcaagtccgccatgcccgaaggc  1260
       A  R  Y  P  D  H  M  K  Q  H  D  F  F  K  S  A  M  P  E  G
1261  tacgtccaggagcgcaccatcttcttcaaggacgacggcaactacaagacccgcgccgag  1320
       Y  V  Q  E  R  T  I  F  F  K  D  D  G  N  Y  K  T  R  A  E
1321  gtgaagttcgagggcgacaccctggtgaaccgcatcgagctgaagggcatcgacttcaag  1380
       V  K  F  E  G  D  T  L  V  N  R  I  E  L  K  G  I  D  F  K
1381  gaggacggcaacatcctggggcacaagcttgagtacaactacaacagccacaacgtctat  1440
       E  D  G  N  I  L  G  H  K  L  E  Y  N  Y  N  S  H  N  V  Y
1441  atcatggccgacaagcagaagaacggcatcaaggtgaacttcaagatccgccacaacatc  1500
       I  M  A  D  K  Q  K  N  G  I  K  V  N  F  K  I  R  H  N  I
1501  gaggacggcagcgtgcagctcgccgaccactaccagcagaacacccccatcggcgacggc  1560
       E  D  G  S  V  Q  L  A  D  H  Y  Q  Q  N  T  P  I  G  D  G
```

```
1561   cccgtgctgctgcccgacaaccactacctgagctaccagtccgccctgagcaaagacccc   1620
        P  V  L  L  P  D  N  H  Y  L  S  Y  Q  S  A  L  S  K  D  P
1621   aacgagaagcgcgatcacatggtcctgctggagttcgtgaccgccgccgggatcactctc   1680
        N  E  K  R  D  H  M  V  L  L  E  F  V  T  A  A  G  I  T  L
1681   ggcatggacgagctgtacaagtaa
        G  M  D  E  L  Y  K  -
```
