# Supplementary material for: MagFRET: The First Genetically Encoded Fluorescent Mg2+ Sensor
Source: PLoS One. 2013 Dec 2;8(12):e82009. doi: 10.1371/journal.pone.0082009 (PMC3846734; doi:10.1371/journal.pone.0082009)
Supplement: Figure S2 — Nucleotide sequence of mammalian expression vector pCMV-MagFRET-1 ORF. The DNA sequence is shown in lowercase, with the single letter amino acid code shown beneath each codon in uppercase. Cerulean is highlighted in turquoise, HsCen3 in red and Citrine in yellow. The two EF-hand motifs are underlined in white. (PDF) [file pone.0082009.s002.pdf]

Figure S2: pCMV-MagFRET-1

```
1     atgggccatatggtgagcaagggcgaggagctgttcaccggggtggtgcccatcctggtc   60
       M  G  H  M  V  S  K  G  E  E  L  F  T  G  V  V  P  I  L  V
61    gagctggacggcgacgtaaacggccacaagttcagcgtgtccggcgagggcgagggcgat   120
       E  L  D  G  D  V  N  G  H  K  F  S  V  S  G  E  G  E  G  D
121   gccacctacggcaagctgaccctgaagttcatctgcaccaccggtaagctgcccgtgccc   180
       A  T  Y  G  K  L  T  L  K  F  I  C  T  T  G  K  L  P  V  P
181   tggcccaccctcgtgaccaccctgacctggggcgtgcagtgcttcgcccgctaccccgac   240
       W  P  T  L  V  T  T  L  T  W  G  V  Q  C  F  A  R  Y  P  D
241   cacatgaagcagcacgacttcttcaagtccgccatgcccgaaggctacgtccaggagcgc   300
       H  M  K  Q  H  D  F  F  K  S  A  M  P  E  G  Y  V  Q  E  R
301   accatcttcttcaaggacgacggcaactacaagacccgcgccgaggtgaagttcgagggc   360
       T  I  F  F  K  D  D  G  N  Y  K  T  R  A  E  V  K  F  E  G
361   gacaccctggtgaaccgcatcgagctgaagggcatcgacttcaaggaggacggcaacatc   420
       D  T  L  V  N  R  I  E  L  K  G  I  D  F  K  E  D  G  N  I
421   ctggggcacaagctggagtacaacgccatcagcgacaacgtctatatcaccgccgacaag   480
       L  G  H  K  L  E  Y  N  A  I  S  D  N  V  Y  I  T  A  D  K
481   cagaagaacggcatcaaggccaacttcaagatccgccacaacatcgaggacggcagcgtg   540
       Q  K  N  G  I  K  A  N  F  K  I  R  H  N  I  E  D  G  S  V
541   cagctcgccgaccactaccagcagaacacccccatcggcgacggccccgtgctgctgccc   600
       Q  L  A  D  H  Y  Q  Q  N  T  P  I  G  D  G  P  V  L  L  P
601   gacaaccactacctgagcacccagtccgccctgagcaaagaccccaacgagaagcgcgat   660
       D  N  H  Y  L  S  T  Q  S  A  L  S  K  D  P  N  E  K  R  D
661   cacatggtcctgctggagttcgtgaccgccgccgggatcactagcgaggaacagaaacaa   720
       H  M  V  L  L  E  F  V  T  A  A  G  I  T  S  E  E  Q  K  Q
721   gaaattaaagatgcttttgaactgtttgatacagacaaagatgaagcaatagattatcat   780
       E  I  K  D  A  F  E  L  F  D  T  D  K  D  E  A  I  D  Y  H
781   gaactgaaggtggcaatgagagccttggggtttgatgtaaaaaaagctgatgtactgaag   840
       E  L  K  V  A  M  R  A  L  G  F  D  V  K  K  A  D  V  L  K
841   attcttaaagattatgacagagaagccacagggaaaatcacctttgaagatttaatgaa   900
       I  L  K  D  Y  D  R  E  A  T  G  K  I  T  F  E  D  F  N  E
901   gttgtgacagactggatattggaaagagatgccatggtgagcaagggcgaggagctgttc   960
       V  V  T  D  W  I  L  E  R  D  A  M  V  S  K  G  E  E  L  F
961   accggggtggtgcccatcctggtcgagctggacggcgacgtaaacggccacaagttcagc   1020
       T  G  V  V  P  I  L  V  E  L  D  G  D  V  N  G  H  K  F  S
1021  gtgtccggcgagggcgagggcgatgccacctacggcaagctgaccctgaagttcatctgc   1080
       V  S  G  E  G  E  G  D  A  T  Y  G  K  L  T  L  K  F  I  C
1081  accaccggcaagctgcccgtgccctggcccaccctcgtgaccaccttcggctacggcctg   1140
       T  T  G  K  L  P  V  P  W  P  T  L  V  T  T  F  G  Y  G  L
1141  atgtgcttcgcccgctaccccgaccacatgaagcagcacgacttcttcaagtccgccatg   1200
       M  C  F  A  R  Y  P  D  H  M  K  Q  H  D  F  F  K  S  A  M
1201  cccgaaggctacgtccaggagcgcaccatcttcttcaaggacgacggcaactacaagacc   1260
       P  E  G  Y  V  Q  E  R  T  I  F  F  K  D  D  G  N  Y  K  T
1261  cgcgccgaggtgaagttcgagggcgacaccctggtgaaccgcatcgagctgaagggcatc   1320
       R  A  E  V  K  F  E  G  D  T  L  V  N  R  I  E  L  K  G  I
1321  gacttcaaggaggacggcaacatcctggggcacaagcttgagtacaactacaacagccac   1380
       D  F  K  E  D  G  N  I  L  G  H  K  L  E  Y  N  Y  N  S  H
1381  aacgtctatatcatggccgacaagcagaagaacggcatcaaggtgaacttcaagatccgc   1440
       N  V  Y  I  M  A  D  K  Q  K  N  G  I  K  V  N  F  K  I  R
1441  cacaacatcgaggacggcagcgtgcagctcgccgaccactaccagcagaacacccccatc   1500
       H  N  I  E  D  G  S  V  Q  L  A  D  H  Y  Q  Q  N  T  P  I
1501  ggcgacggccccgtgctgctgcccgacaaccactacctgagctaccagtccgccctgagc   1560
       G  D  G  P  V  L  L  P  D  N  H  Y  L  S  Y  Q  S  A  L  S
```

```
1561   aaagaccccaacgagaagcgcgatcacatggtcctgctggagttcgtgaccgccgccggg   1620
        K  D  P  N  E  K  R  D  H  M  V  L  L  E  F  V  T  A  A  G
1621   atcactctcggcatggacgagctgtacaagtaa
        I  T  L  G  M  D  E  L  Y  K  -
```
