# Supplementary material for: MagFRET: The First Genetically Encoded Fluorescent Mg2+ Sensor
Source: PLoS One. 2013 Dec 2;8(12):e82009. doi: 10.1371/journal.pone.0082009 (PMC3846734; doi:10.1371/journal.pone.0082009)
Supplement: Figure S3 — Nucleotide sequence of mammalian expression vector pCMV-MagFRET-1-NLS ORF. The DNA sequence is shown in lowercase, with the single letter amino acid code shown beneath each codon in uppercase. Cerulean is highlighted in turquoise, HsCen3 in red, Citrine in yellow and the three PKKKRKV repeats in grey. The two EF-hand motifs are underlined in white. (PDF) [file pone.0082009.s003.pdf]

Figure S3: pCMV-MagFRET-1-NLS

```
   1  atgggccatatggtgagcaagggcgaggagctgttcaccggggtggtgcccatcctggtc  60
       M  G  H  M  V  S  K  G  E  E  L  F  T  G  V  V  P  I  L  V
  61  gagctggacggcgacgtaaacggccacaagttcagcgtgtccggcgagggcgagggcgat  120
       E  L  D  G  D  V  N  G  H  K  F  S  V  S  G  E  G  E  G  D
 121  gccacctacggcaagctgaccctgaagttcatctgcaccaccggtaagctgcccgtgccc  180
       A  T  Y  G  K  L  T  L  K  F  I  C  T  T  G  K  L  P  V  P
 181  tggcccaccctcgtgaccaccctgacctggggcgtgcagtgcttcgcccgctaccccgac  240
       W  P  T  L  V  T  T  L  T  W  G  V  Q  C  F  A  R  Y  P  D
 241  cacatgaagcagcacgacttcttcaagtccgccatgcccgaaggctacgtccaggagcgc  300
       H  M  K  Q  H  D  F  F  K  S  A  M  P  E  G  Y  V  Q  E  R
 301  accatcttcttcaaggacgacggcaactacaagacccgcgccgaggtgaagttcgagggc  360
       T  I  F  F  K  D  D  G  N  Y  K  T  R  A  E  V  K  F  E  G
 361  gacaccctggtgaaccgcatcgagctgaagggcatcgacttcaaggaggacggcaacatc  420
       D  T  L  V  N  R  I  E  L  K  G  I  D  F  K  E  D  G  N  I
 421  ctggggcacaagctggagtacaacgccatcagcgacaacgtctatatcaccgccgacaag  480
       L  G  H  K  L  E  Y  N  A  I  S  D  N  V  Y  I  T  A  D  K
 481  cagaagaacggcatcaaggccaacttcaagatccgccacaacatcgaggacggcagcgtg  540
       Q  K  N  G  I  K  A  N  F  K  I  R  H  N  I  E  D  G  S  V
 541  cagctcgccgaccactaccagcagaacacccccatcggcgacggccccgtgctgctgccc  600
       Q  L  A  D  H  Y  Q  Q  N  T  P  I  G  D  G  P  V  L  L  P
 601  gacaaccactacctgagcacccagtccgccctgagcaaagaccccaacgagaagcgcgat  660
       D  N  H  Y  L  S  T  Q  S  A  L  S  K  D  P  N  E  K  R  D
 661  cacatggtcctgctggagttcgtgaccgccgccgggatcactagcgaggaacagaaacaa  720
       H  M  V  L  L  E  F  V  T  A  A  G  I  T  S  E  E  Q  K  Q
 721  gaaattaaagatgcttttgaactgtttgatacagacaaagatgaagcaatagattatcat  780
       E  I  K  D  A  F  E  L  F  D  T  D  K  D  E  A  I  D  Y  H
 781  gaactgaaggtggcaatgagagccttggggtttgatgtaaaaaaagctgatgtactgaag  840
       E  L  K  V  A  M  R  A  L  G  F  D  V  K  K  A  D  V  L  K
 841  attcttaaagattatgacagagaagccacagggaaaatcacctttgaagatttaatgaa  900
       I  L  K  D  Y  D  R  E  A  T  G  K  I  T  F  E  D  F  N  E
 901  gttgtgacagactggatattggaaagagatgccatggtgagcaagggcgaggagctgttc  960
       V  V  T  D  W  I  L  E  R  D  A  M  V  S  K  G  E  E  L  F
 961  accggggtggtgcccatcctggtcgagctggacggcgacgtaaacggccacaagttcagc  1020
       T  G  V  V  P  I  L  V  E  L  D  G  D  V  N  G  H  K  F  S
1021  gtgtccggcgagggcgagggcgatgccacctacggcaagctgaccctgaagttcatctgc  1080
       V  S  G  E  G  E  G  D  A  T  Y  G  K  L  T  L  K  F  I  C
1081  accaccggcaagctgcccgtgccctggcccaccctcgtgaccaccttcggctacggcctg  1140
       T  T  G  K  L  P  V  P  W  P  T  L  V  T  T  F  G  Y  G  L
1141  atgtgcttcgcccgctaccccgaccacatgaagcagcacgacttcttcaagtccgccatg  1200
       M  C  F  A  R  Y  P  D  H  M  K  Q  H  D  F  F  K  S  A  M
1201  cccgaaggctacgtccaggagcgcaccatcttcttcaaggacgacggcaactacaagacc  1260
```

```
       P  E  G  Y  V  Q  E  R  T  I  F  F  K  D  D  G  N  Y  K  T
1261   cgcgccgaggtgaagttcgagggcgacaccctggtgaaccgcatcgagctgaagggcatc   1320
       R  A  E  V  K  F  E  G  D  T  L  V  N  R  I  E  L  K  G  I
1321   gacttcaaggaggacggcaacatcctggggcacaagcttgagtacaactacaacagccac   1380
       D  F  K  E  D  G  N  I  L  G  H  K  L  E  Y  N  Y  N  S  H
1381   aacgtctatatcatggccgacaagcagaagaacggcatcaaggtgaacttcaagatccgc   1440
       N  V  Y  I  M  A  D  K  Q  K  N  G  I  K  V  N  F  K  I  R
1441   cacaacatcgaggacggcagcgtgcagctcgccgaccactaccagcagaacacccccatc   1500
       H  N  I  E  D  G  S  V  Q  L  A  D  H  Y  Q  Q  N  T  P  I
1501   ggcgacggccccgtgctgctgcccgacaaccactacctgagctaccagtccgccctgagc   1560
       G  D  G  P  V  L  L  P  D  N  H  Y  L  S  Y  Q  S  A  L  S
1561   aaagaccccaacgagaagcgcgatcacatggtcctgctggagttcgtgaccgccgccggg   1620
       K  D  P  N  E  K  R  D  H  M  V  L  L  E  F  V  T  A  A  G
1621   atcactctcggcatggacgagctgtacaagggcggatctcccaagaaaaaacgcaaggtg   1680
       I  T  L  G  M  D  E  L  Y  K  G  G  S  P  K  K  K  R  K  V
1681   gatccaaagaaaaagcgtaaagttgatccaaaaaagaagagaaaggtagatctataa
       D  P  K  K  K  R  K  V  D  P  K  K  K  R  K  V  D  L  -
```
