# Supplementary figures and images for: MagFRET: The First Genetically Encoded Fluorescent Mg2+ Sensor
Source: PLoS One. 2013 Dec 2;8(12):e82009. doi: 10.1371/journal.pone.0082009 (PMC3846734; doi:10.1371/journal.pone.0082009)

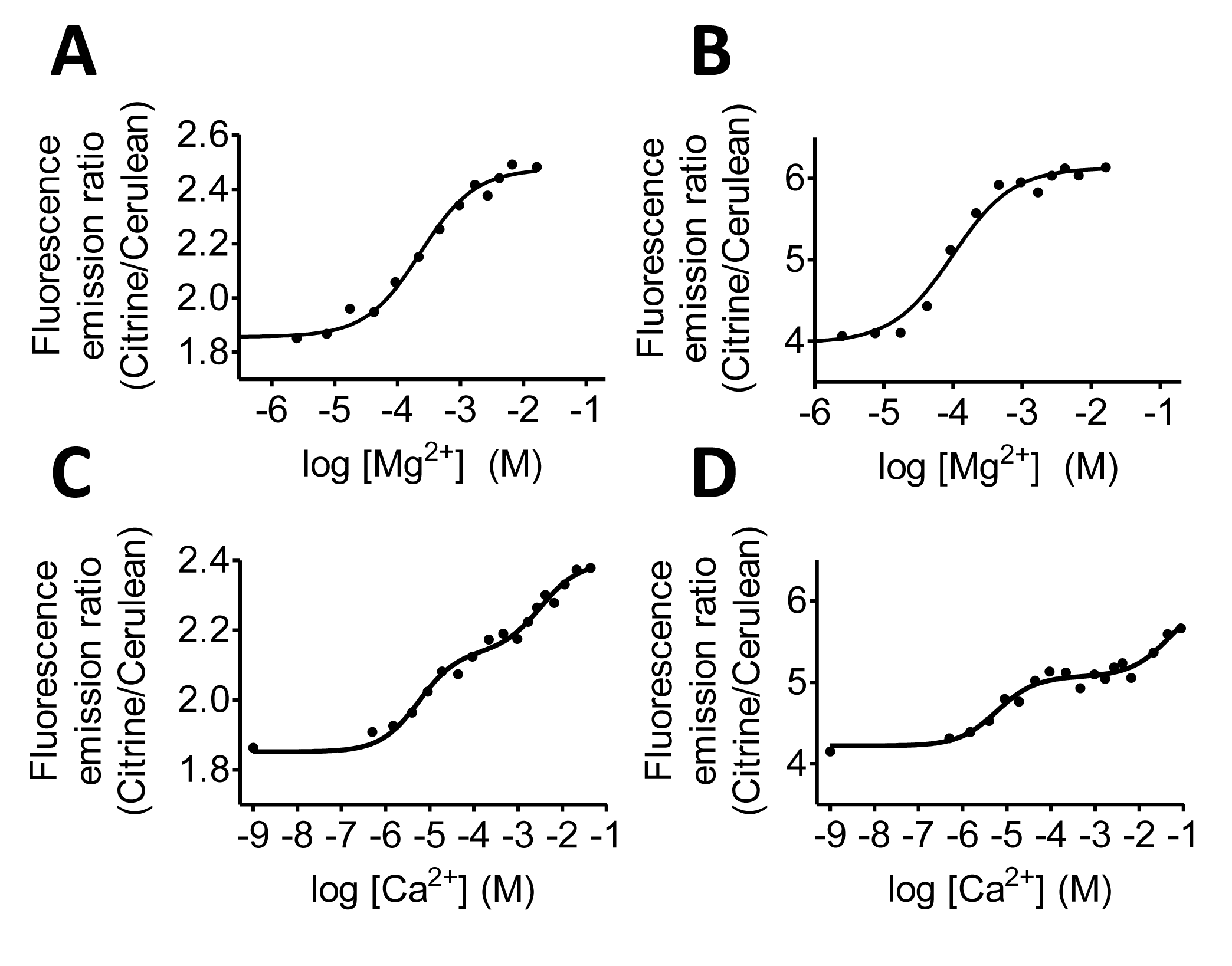

Supplement: Figure S4 — Effect of pH on MagFRET-1. To check for pH sensitivity, the MagFRET-1 emission ratio was followed as a function of Mg2+ (A, B) and Ca2+ (C, D) concentration, at pH 6 (A, C) and pH 8 (B, D). Fitting of the data revealed a MagFRET-1 K d for Mg2+ of 230±35 µM at pH 6 and 99±18 µM at pH 8. The sensor's K d for Ca2+ (first binding event) at pH = 6 was found to be 5.6±1.7 µM, while at pH 8 it was 5.9±1.9 µM. Buffers used were 150 mM MES (pH 6), 100 mM NaCl and 10% glycerol for pH 6 and 150 mM Tris (pH 8), 100 mM NaCl and 10% glycerol for pH 8. (TIF) [file pone.0082009.s004.tif]

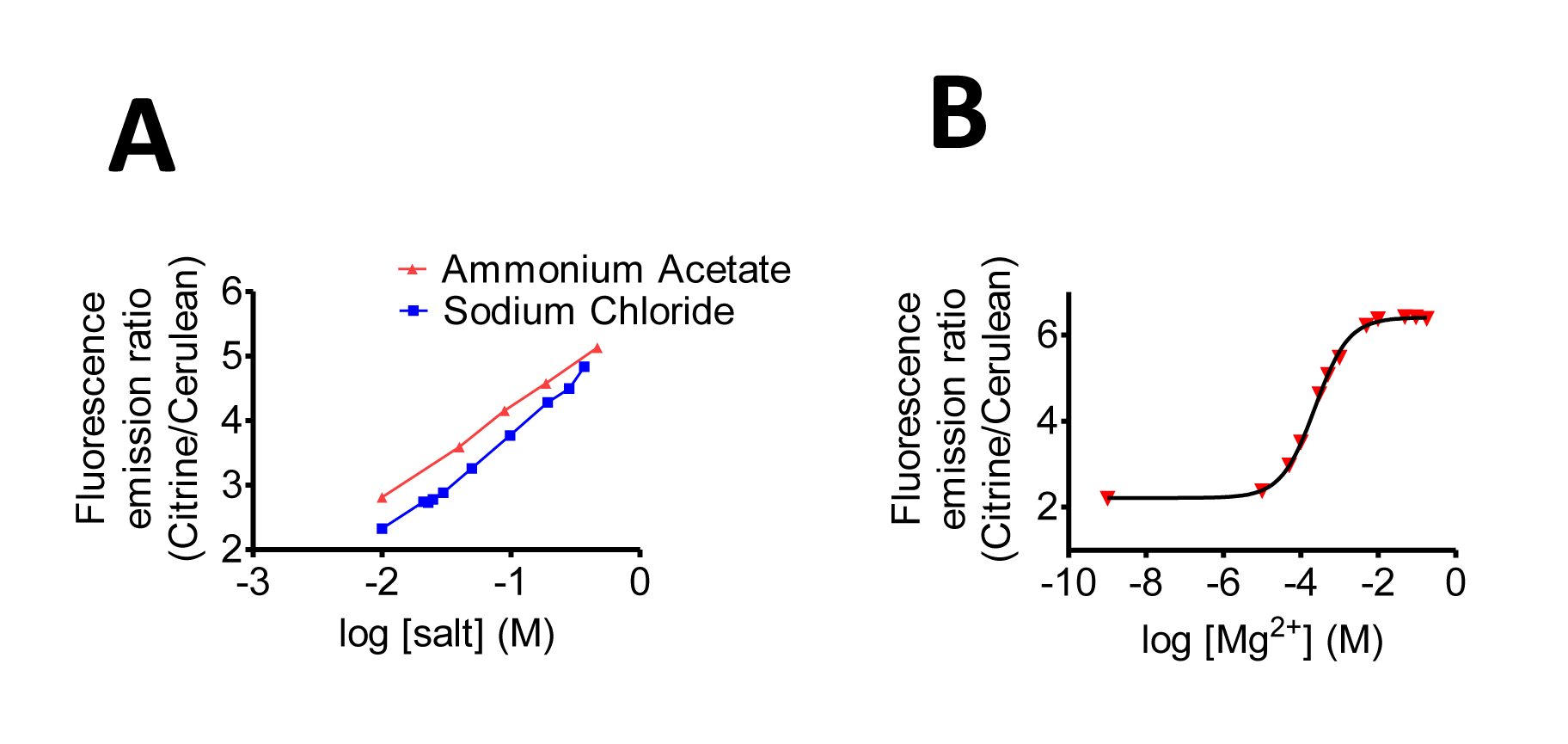

Supplement: Figure S5 — Effect of ionic strength on MagFRET-1. (A) Emission ratio of MagFRET-1 at increasing concentrations of ammonium acetate or NaCl in a buffer with low ionic strength. (B) Emission ratio of MagFRET-1 as a function of Mg2+ concentration in a buffer with low ionic strength. The low ionic strength buffer used in (A, B) was 20 mM Hepes (pH 7.1), 10 mM NaCl, 10% (v/v) glycerol. Fitting of the data using a single binding event revealed a K d for Mg2+ of 231±10 µM. (TIF) [file pone.0082009.s005.tif]

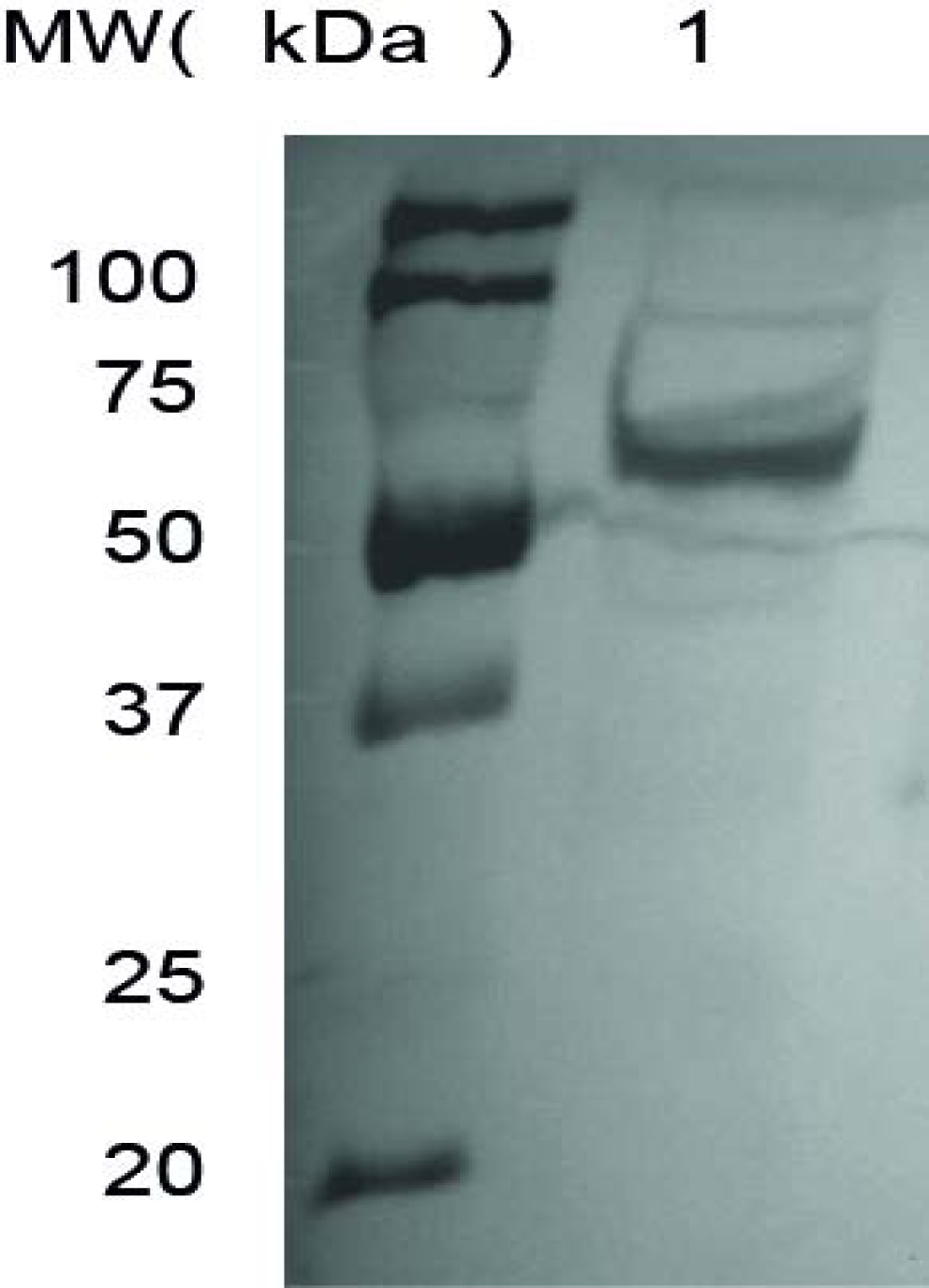

Supplement: Figure S6 — Western blot analysis of MagFRET-1 expressing HEK293 cells. A molecular weight marker (Precision Plus Protein Standards, Bio-Rad) was loaded in the left-hand lane. Lane 1 displays the lysate of HEK293 cells transfected with a vector encoding for MagFRET-1 under control of a CMV promoter. The blotting membrane was incubated with mouse anti-GFP (Ab3277, Abcam), followed by HRP-functionalized goat anti-mouse antibody (Dako). The calculated molecular weight for MagFRET-1 is 62 kDa. (TIF) [file pone.0082009.s006.tif]

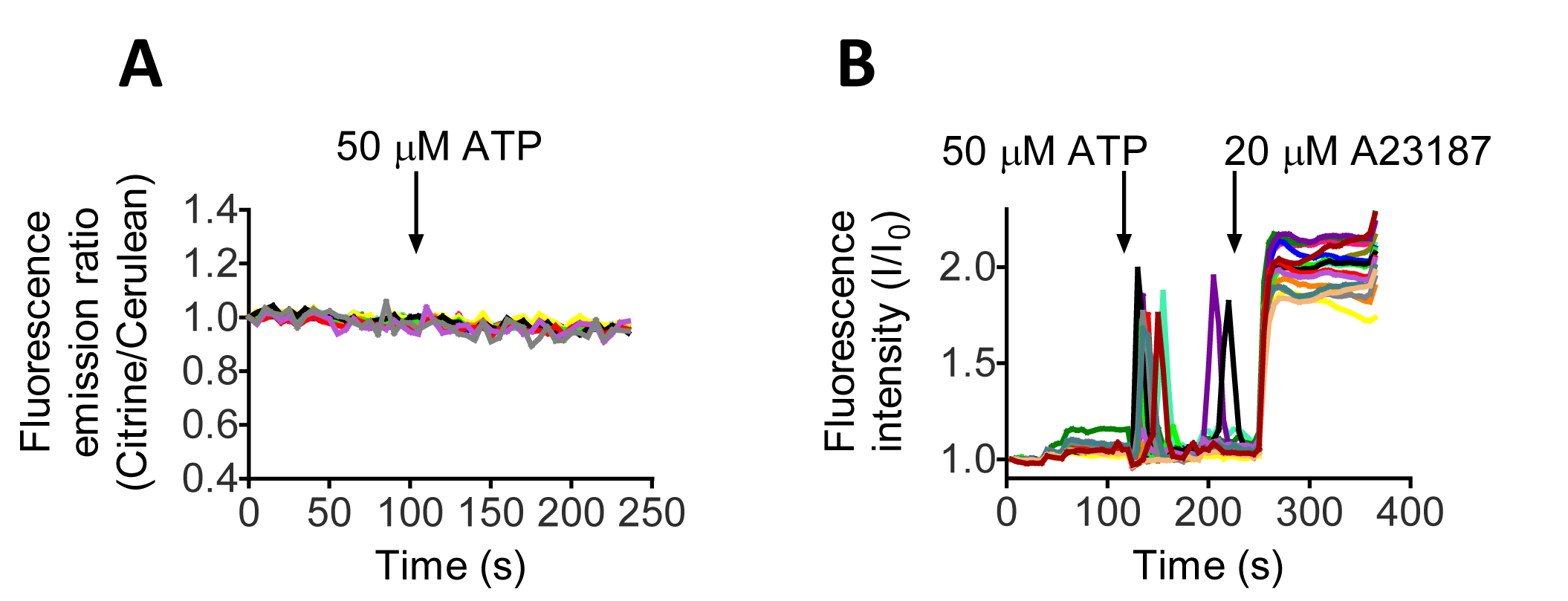

Supplement: Figure S7 — Investigation of MagFRET-1 response to elevated cytosolic Ca2+ induced by ATP. (A) Emission ratio over time of intact HEK293 cells expressing MagFRET-1 measured by widefield fluorescence microscopy. At t = 104 s, 50 µM ATP was added to activate Ca2+ signaling. (B) To confirm Ca2+ signaling took place in stimulated cells, the fluorescence intensity of intact HEK293 cells loaded with Ca2+-dye Oregon Green–BAPTA was followed. At t = 104 s, 50 µM ATP was added to activate Ca2+ signaling, and at t = 226 s, 20 µM of the Ca2+ ionophore A23187 was added. In A and B, each trace represents the response of an individual cell, with ratio (A) or intensity (B) normalized to the value at t = 0 s. (TIF) [file pone.0082009.s007.tif]
