# Supplementary material for: MagFRET: The First Genetically Encoded Fluorescent Mg2+ Sensor
Source: PLoS One. 2013 Dec 2;8(12):e82009. doi: 10.1371/journal.pone.0082009 (PMC3846734; doi:10.1371/journal.pone.0082009)
Supplement: Method S1 — Western blotting. (PDF) [file pone.0082009.s008.pdf]

Method S1: Western blotting. HEK293 cells transfected with MagFRET-1 were kept on ice, washed 3 times with PBS and subsequently lysed in 100 µL of PBS containing 1 mM EDTA, 1 % (v/v) Igepal® CA630 (Sigma) and 1x complete inhibitor protease cocktail (Roche). Following lysis, cells were transferred to an eppendorf tube and incubated for 30 minutes on ice. Next, gel samples (20 µL) were made and loaded on a 12% SDS-PAGE gel, together with a molecular weight marker (Precision Plus Protein Standards, Bio-Rad). The protein was transferred to a nitrocellulose membrane, followed by blocking overnight using 5% skimmed milk in TBS-T and 1 h incubation of mouse anti-GFP (Ab3277, AbCam) (1:2000) in the same buffer. HRP functionalized goat anti-mouse (1:5000, Dako) was used as a secondary antibody. Proteins were visualized using a tetramethylbenzidine (TMB) solution (Pierce) according to manufacturers' instructions.
