# Supplementary material for: MagFRET: The First Genetically Encoded Fluorescent Mg2+ Sensor
Source: PLoS One. 2013 Dec 2;8(12):e82009. doi: 10.1371/journal.pone.0082009 (PMC3846734; doi:10.1371/journal.pone.0082009)
Supplement: Table S1 — Primers used for mutagenesis of HsCen3. (PDF) [file pone.0082009.s009.pdf]

Table S1. Primers Used for Mutagenesis of HsCen3.

MagFRET variant	Site directed mutagenesis primers (5'→3')
MagFRET-2	GCCACCTTCAGTTCATGATAATCTATTGCaTCATCTTTGTCTGTATC
MagFRET-3	GCTTATCTTTGTCTGTtTCAAACAGTTCAAAAGCATC
MagFRET-4	GCCACCTTCAGTTCATGATAATCTATTGCTTCtTCTTTGTCTGTATC
MagFRET-5	GCCACCTTCAGTTCATGATAATCTATgtCTTCATCTTTGTCTGTATC
MagFRET-6	GCCACCTTCAGTTCATGATAATCTATTtCTTCtTCTTTGTCTGTATC
MagFRET-7	Forward: GACAGAGAAGCCACAAAGAAAATCACCTTTGAAG
	Reverse: CTTCAAAGGTGATTTTCTTTGTGGCTTCTCTGTC
MagFRET-8	Forward: CTGAAGATTCTTAAAGATTATAAGAGAGAAGCCACAGGG
	Reverse: CCCTGTGGCTTCTCTCTTATAATCTTTAAGAATCTTCAG
